# Incorporation of *N7*-Platinated Guanines into Thermus Aquaticus (Taq) DNA Polymerase: Atomistic Insights from Molecular Dynamics Simulations

**DOI:** 10.3390/ijms24129849

**Published:** 2023-06-07

**Authors:** Federica De Castro, Giada Ciardullo, Francesco Paolo Fanizzi, Mario Prejanò, Michele Benedetti, Tiziana Marino

**Affiliations:** 1 Dipartimento di Scienze e Tecnologie Biologiche ed Ambientali, Università del Salento, Prov.le Lecce-Monteroni, Centro Ecotekne, I-73100 Lecce, Italy; federica.decastro@unisalento.it (F.D.C.); fp.fanizzi@unisalento.it (F.P.F.); 2 Dipartimento di Chimica e Tecnologie Chimiche, Laboratorio PROMOCS cubo 14C, Università della Calabria, I-87036 Rende, Italy; giada.ciardullo@unical.it (G.C.); mario.prejano@unical.it (M.P.)

**Keywords:** nucleoside analogues, platinum compounds, coordination compounds, cisplatin, antitumor drugs, antiviral drugs, antimetabolites, molecular dynamics

## Abstract

In this work, we elucidated some key aspects of the mechanism of action of the cisplatin anticancer drug, *cis*-[Pt(NH_3_)_2_Cl_2_], involving direct interactions with free nucleotides. A comprehensive in silico molecular modeling analysis was conducted to compare the interactions of Thermus aquaticus (Taq) DNA polymerase with three distinct *N7*-platinated deoxyguanosine triphosphates: [Pt(dien)(*N7*-dGTP)] (**1**), *cis*-[Pt(NH_3_)_2_Cl(*N7*-dGTP)] (**2**), and *cis*-[Pt(NH_3_)_2_(H_2_O)(*N7*-dGTP)] (**3**) {dien = diethylenetriamine; dGTP = 5′-(2′-deoxy)-guanosine-triphosphate}, using canonical dGTP as a reference, in the presence of DNA. The goal was to elucidate the binding site interactions between Taq DNA polymerase and the tested nucleotide derivatives, providing valuable atomistic insights. Unbiased molecular dynamics simulations (200 ns for each complex) with explicit water molecules were performed on the four ternary complexes, yielding significant findings that contribute to a better understanding of experimental results. The molecular modeling highlighted the crucial role of a specific α-helix (O-helix) within the fingers subdomain, which facilitates the proper geometry for functional contacts between the incoming nucleotide and the DNA template needed for incorporation into the polymerase. The analysis revealed that complex **1** exhibits a much lower affinity for Taq DNA polymerase than complexes **2**–**3**. The affinities of cisplatin metabolites **2**–**3** for Taq DNA polymerase were found to be quite similar to those of natural dGTP, resulting in a lower incorporation rate for complex **1** compared to complexes **2**–**3**. These findings could have significant implications for the cisplatin mechanism of action, as the high intracellular availability of free nucleobases might promote the competitive incorporation of platinated nucleotides over direct cisplatin attachment to DNA. The study’s insights into the incorporation of platinated nucleotides into the Taq DNA polymerase active site suggest that the role of platinated nucleotides in the cisplatin mechanism of action may have been previously underestimated.

## 1. Introduction

Metal-based drugs offer a broad spectrum of therapeutic applications and are considered state-of-the-art treatments for numerous diseases [1]. Well-known examples include platinum complexes widely used in cancer treatment [2,3] and gold complexes used for rheumatoid arthritis [4,5]. It is also important to mention the important role of drugs based on arsenic (for treating trypanosomiasis [6]), antimony (for addressing leishmaniasis [7,8]), bismuth (for combating antibiotic-resistant bacteria [9]), and lithium compounds (for the management of neurological and cardiovascular disorders [10]). The wide range of metals and relative compounds characterized by useful pharmacological activity suggests that many other new and innovative applications of metal-based drugs are currently waiting to be discovered [2,11,12,13,14,15,16].

Focusing our attention on platinum-based drugs, it can be noted that the spectrum of coordination compounds, including organometallic species, designed to be studied for antitumor activity has been strongly increased in the last decades [17,18,19,20,21,22,23,24]. Notwithstanding this aspect, nowadays the most common platinum drugs remain cisplatin, carboplatin, and oxaliplatin, which are utilized in about 50% of cancer chemotherapies. These drugs are effective on a wide spectrum of tumor diseases because they are able to disrupt DNA replication and transcription, giving rise to different types of platinum-DNA adducts and crosslinks, which induce cell death through apoptosis [2,25,26,27]. It is noteworthy that even for these successful drugs, there is a need for new active complexes to overcome the intrinsic or acquired resistance of some tumors, while reducing the relevant side effects [2,24]. Interestingly, some tested platinum compounds also show clear antiviral activity [11].

To better contextualize this work, it is useful to focus our attention on another successful class of antitumor/antiviral drugs, nucleos(t)ide analogs (NAs). Common nucleos(t)ide antimetabolites are structurally similar to natural nucleos(t)ides and are widely used in the treatment of cancer/viral diseases [28,29,30,31,32,33,34,35,36]. Developed over the past fifty years, NAs can interfere with nucleic acid production and functionality, thereby impeding cancer cells’/viruses’ growth and survival [37,38]. Novel NAs have been developed to target specific biological processes through various structural and chemical modifications of the natural nucleotides [39]. These compounds typically enter cells via plasma membrane nucleoside transporters and undergo phosphorylation to generate the active triphosphate forms [32,40,41]. In this case, competition with natural counterparts inhibits essential enzymes and disrupts cancer cells’ growth and/or viral replication [32]. Resistance phenomena to NAs drugs can arise from various factors, including inefficient uptake and faulty apoptosis induction [42,43]. This drug family is now considered essential for creating innovative drugs capable of combating new epidemic viruses [28,33,41,44,45,46,47]. The related research activities were boosted by the pandemic worldwide diffusion of pathogenic viruses (HIV, hepatitis, SARS, etc.), producing the approval of many new protocols for the treatment of viral diseases, including NAs [31,48].

We believe that the development of novel drug species based on NAs and other chemotherapeutic agents could improve the treatment outcomes for many tumor/viral pathologies [49,50]. Indeed, platinated nucleos(t)ides have the potential to combine in the same molecule the pharmacological properties of both nucleos(t)ide analogs and platinum-based drugs. In other words, these molecules hold the promise to be a new class of antitumor/antiviral antimetabolites, products of the merging of the two classes of platinum-based drugs and modified NAs. The suitability of this approach was confirmed by some previously reported studies evaluating the antitumor/antiviral properties of platinum-nucleos(t)ide compounds [11,51,52,53,54,55,56,57,58,59,60,61,62].

**Figure 1:**
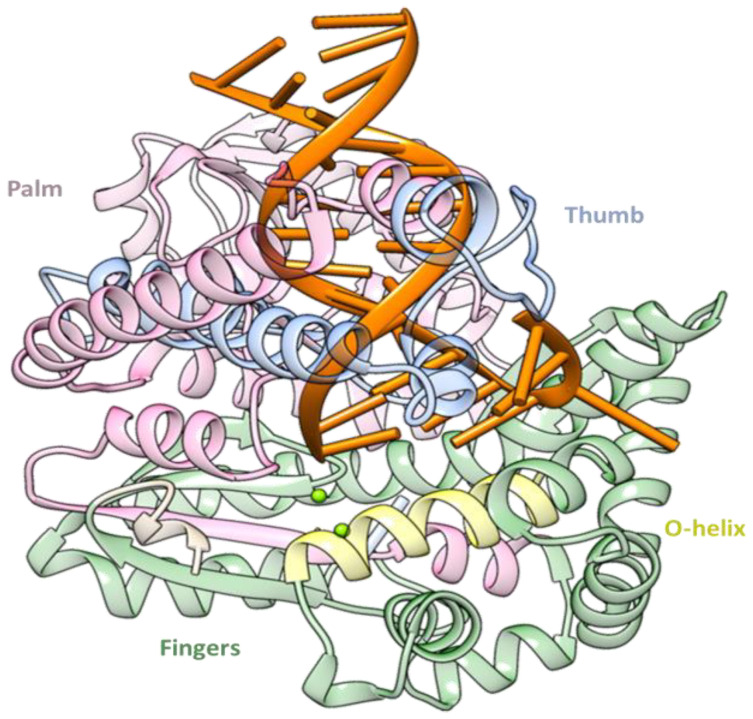
Structure of the Thermus Aquaticus DNA Polymerase enzyme in complex with DNA (PDB code 5YTD) [65].

**Scheme 1:**
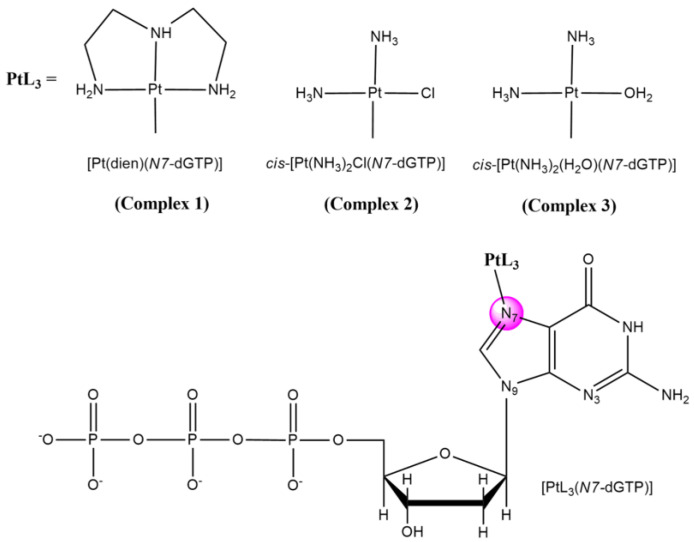
Schematic representation of complexes: [Pt(dien)(*N7*-dGTP)] (**1**), *cis*-[Pt(NH_3_)_2_Cl(*N7*-dGTP)] (**2**), and *cis*-[Pt(NH_3_)_2_(H_2_O)(*N7*-dGTP)] (**3**); dGTP = 5′-(2′-deoxy)-guanosine triphosphate. They are generally indicated in short as [PtL_3_(*N7*-dGTP)] on the bottom, together with the relative Pt-containing moieties under investigation, indicated on the top.

In our previous studies, we discovered that Thermus Aquaticus (Taq) and Mitochondrial γ DNA polymerases (see Figure 1) can recognize and incorporate the model [Pt(dien)(*N7*-dGTP)], complex **1** {dien = diethylenetriamine; dGTP = 5′-(2′-deoxy)guanosine triphosphate, see Scheme 1}, into synthesized DNA [59,60,61], as is generally the case for nucleos(t)ide analogue-based drugs [48]. This enables site-specific metalation, interference with the metabolism of nucleic acids, and the potential for pharmacological effects [59,61,63].

In our previous studies, we also considered the interaction of the model [Pt(dien)(*N7*-GTP)] (GTP = 5′-guanosine triphosphate) complex with the model T7 RNA polymerase. However, it was more selective than the previously tested DNA polymerases and unable to incorporate the platinated GTP [64]. This suggests that platinated deoxyribonucleotides could be used to specifically target DNA without affecting RNA. This opportunity probably stems from the well-known higher activation energy and selectivity observed for NTPs over dNTPs during insertion into their specific substrates [64].

This work aims to study the potential pharmacological applications of metalated nucleos(t)ides. Generally, for nucleos(t)ide analogues, the mechanism of incorporation into DNA polymerases, which produces the observed final insertion into DNA, is investigated [61,63,66,67,68,69,70,71,72,73,74,75]. For this study, we employed Molecular Dynamics Simulations to gain atomistic insights into the mechanism of incorporation of *N7*-platinated guanines in complexes of the types [Pt(dien)(*N7*-dGTP)] (**1**), *cis*-[Pt(NH_3_)_2_Cl(*N7*-dGTP)] (**2**), and *cis*-[Pt(NH_3_)_2_(H_2_O)(*N7*-dGTP)] (**3**) (see Scheme 1), into the model Taq DNA Polymerase. We choose to consider both Cl^−^- and H_2_O-containing species of the platinated base since no clear indications are available on the possible forms interacting with the nucleic acid targets.

This investigation is worthwhile because the mechanism of incorporation is an essential step for the metabolic interactions determining the occurrence of antitumor/antiviral activity and needs further clarification. A more detailed understanding of the incorporation mechanism of metalated purines into DNA polymerases is crucial for the optimized design of new analogues that can effectively interfere with the activity of DNA polymerases. Indeed, these latter constitute key targets as essential cellular and viral “machines”. Specific incorporation studies could also disclose alternative processes of DNA platination in cells treated with cisplatin, *cis*-[Pt(NH_3_)_2_Cl_2_], and related antitumor drugs involving previously platinated nucleotides and DNA polymerases. This may occur in parallel with direct DNA platination processes. Moreover, incorporation studies are also profitably linked to the optimization of the platinum-modified nucleotide structure, which could enhance the interactions with tumor cells and viral DNA polymerases, generating higher antitumor/antiviral activity.

## 2. Results and Discussion

In this work, we studied model complexes based on purine nucleotides *N7*-coordinated to a platinum center, working as modified nucleotides with unaltered sugar moieties and the indicated electric charge calculated at physiological pH (7.4), depicted in Scheme 1. Complex **1** was found to be a possible substrate for human cell membrane transporters in the HeLa cervical cancer cell model [57] and rat liver mitochondrial membrane transporters [58,59]. This was also found to be recognized by Taq and γ DNA polymerases, with the consequent possibility of being incorporated into newly synthesized DNA [59,61,63]. Therefore, in this study, we focused on the structural behavior observed within the active site cleft of Taq:DNA polymerase and its neighboring area when interacting with the considered **1**–**3** platinated nucleotides shown in Scheme 1. For comparison, MD simulations were also extended to the Taq-DNA polymerase in the presence of the natural nucleotide dGTP.

**Figure 2:**
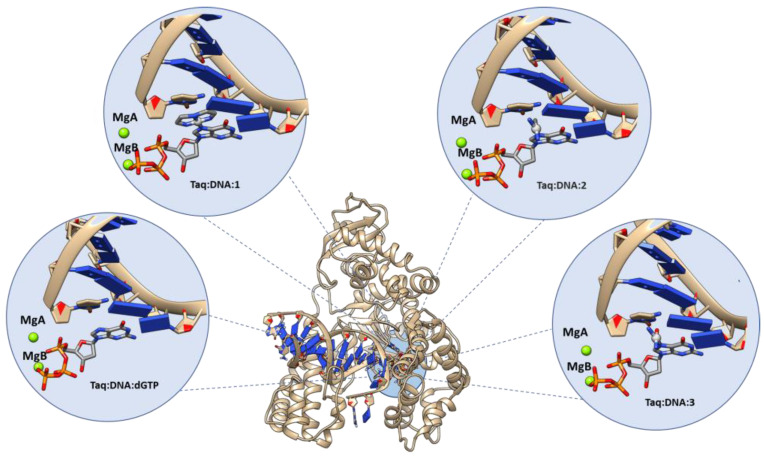
The four considered different Taq:DNA:substrate systems: Taq:DNA:dGTP, Taq:DNA:**1**, Taq:DNA:**2** and Taq:DNA:**3**; {**1** = [Pt(dien)(*N7*-dGTP)]; **2** = *cis*-[Pt(NH_3_)_2_Cl(*N7*-dGTP)]; **3** = *cis*-[Pt(NH_3_)_2_(H_2_O)(*N7*-dGTP)]}.

**Figure 3:**
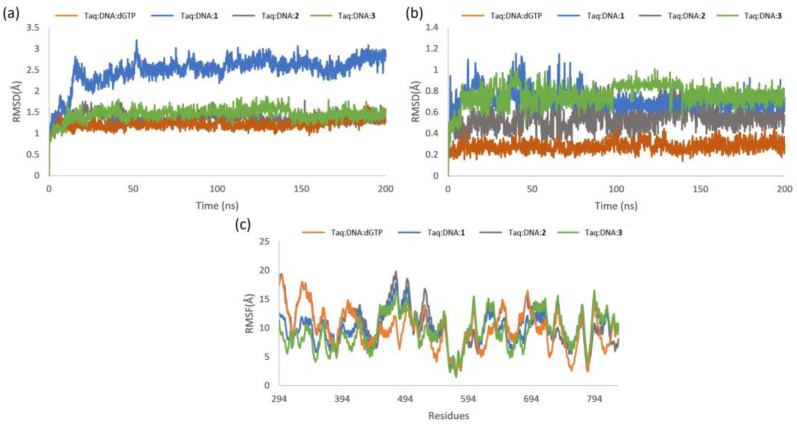
Root mean square deviation (RMSD) of (**a**) backbone atoms of the protein and (**b**) atoms of deoxyribonucleotide triphosphate (dNTP) bases, and (**c**) root mean square fluctuation (RMSF) calculated for backbone atoms of different Taq:DNA:substrate systems {**1** = [Pt(dien)(*N7*-dGTP)]; **2** = *cis*-[Pt(NH_3_)_2_Cl(*N7*-dGTP)]; **3** = *cis*-[Pt(NH_3_)_2_(H_2_O)(*N7*-dGTP)]}.

In the starting structure, the dNTPs of all the ternary complexes (Figure 2) are similarly oriented, featuring the triphosphate moiety towards Mg^2+^ ions, so that, in principle, the base is prone to pair with the natural base cytosine of the template. Mg^2+^ ions are important due to the stabilization of the negative charge concentrated in the active site during nucleotide addition. Mg^2+^ occupying “site A” is involved in the coordination of the alpha phosphate (Pα) of the dNTP and the hanging 3′-OH, while the other one occupying “site B” coordinates with the Pβ and Pγ of the dNTP and two nearby aspartate residues of the Taq-DNA polymerase. To evaluate the structural stability over the MD trajectories, we examined the RMSDs of the Cα atoms of the protein backbone in all the investigated ternary complexes using data arising from one MD trajectory per system (Figure 3a).

The RMSD analysis indicates that these systems are not fully equilibrated until ∼15 ns. Thereafter, the RMSD fluctuations observed in the trajectory remain within 1.5 Å for dGTP, **2**, and **3**, indicating that these are reasonably stable structures. In the case of **1**, however, the RMSD values fluctuate within 2.5 Å, indicating a major variability during the simulation time compared to the other systems. This behavior is not attributable specifically to the platinated moiety, since Figure 3b also reports the RMSD values related to the dNTP moiety for each investigated system. It is evident that a very similar trend characterizes all three platinated nucleotides compared to the natural dGTP, with RMSD fluctuations within the trajectory of each platinated nucleotide of about 0.7 Å with respect to dGTP (0.2 Å). This result confirms that the presence of the platinated moiety does not lead to any “extra” evident structural organization on the Taq-DNA polymerase, but just on the nucleotide counterpart.

The root-mean-square fluctuation (RMSF) of the Cα atoms was calculated to locate the more flexible regions of the three systems containing platinated nucleotides with respect to those with the natural dGTP (see Figure 2c). It is clear that the most evident mobility is found in the region, including the 294–394 residues very close to the thumb domain, particularly for the Taq:DNA:dGTP ternary complex. This is mainly related to the solvent-protein interactions of the terminal region of the Taq enzyme, as confirmed by visual inspection of the principal component analysis (see Figure S1).

**Figure 4:**
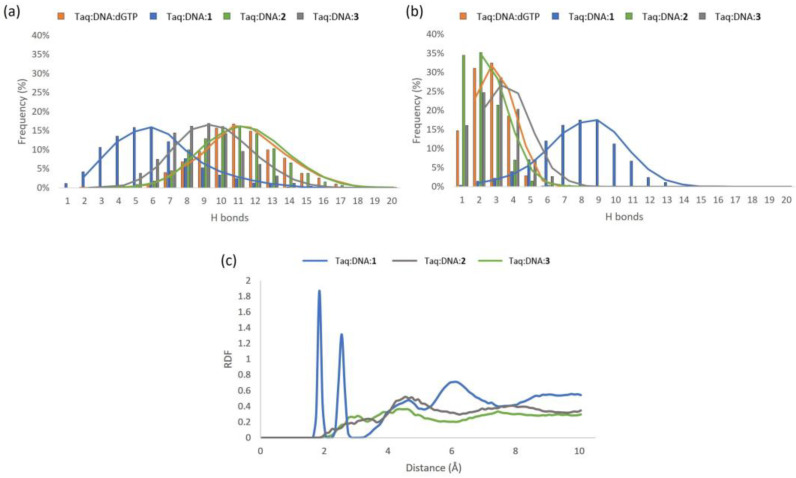
Number of hydrogen-bond interactions involving the dNTP species and (**a**) protein, (**b**) water molecules, and (**c**) radial distribution functions calculated for the dNTP-H_2_O pair {**1** = [Pt(dien)(*N7*-dGTP)]; **2** = *cis*-[Pt(NH_3_)_2_Cl(*N7*-dGTP)]; **3** = *cis*-[Pt(NH_3_)_2_(H_2_O)(*N7*-dGTP)]}.

The analysis of the radius of gyration and of the superposition of the 10 representative structures collected during the simulations further revealed a stable compactness reached by the four systems (see Figure S2), while details on the hierarchical clustering are present in Table S1. Analyzing the data on the H-bonds within 5 Å for the distances between donor and acceptor and 20° for their angle (Figure 4a,b) reveals that **2** and **3** show more similar behavior to that of dGTP compared to **1**. This platinated species indeed exhibits a different topological nature in the dNTP binding site, both regarding the H-bonds with the protein counterpart (Figure 4a) and those with the solvent (Figure 4b). It is more involved in establishing H-bonds, with water molecules acting as both donor and acceptor, unlike the other two platinated nucleotides.

This finding is consistent with fewer H-bonds with the protein, indicating that **1** allows access to a greater number of water molecules into the catalytic pocket compared to the other two platinated nucleotides.

**Figure 5:**
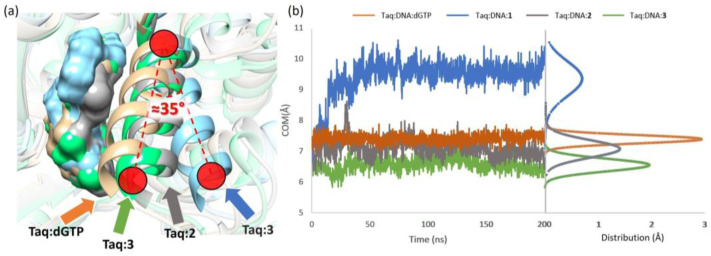
(**a**) Superposition of the four investigated systems, with focus on the α-helix dedicated to recognition, and (**b**) distances between the centers of mass (COM) of the four different dNTP and the α-helix (relative distribution of distances is also shown), {where: **1** = [Pt(dien)(*N7*-dGTP)]; **2** = *cis*-[Pt(NH_3_)_2_Cl(*N7*-dGTP)]; **3** = *cis*-[Pt(NH_3_)_2_(H_2_O)(*N7*-dGTP)]}.

The water accessibility was also detected from the water radial distribution function (RDF) analysis (see Figure 4c), which was obtained as a function of the distance between the water oxygen and the platinated moiety of **1**, **2**, and **3** species. From Figure 4c, it can be noted that a different water distribution is present since clear solvation shells are achievable in the case of **1** starting from a distance of less than 2 Å, while in the cases of **2** and **3**, only solvent bulk is notable. A similar behavior is observed in the RDF as a function of the distance between the water oxygen and each nitrogen atom of the platinated moiety (Figure S3), which underlines a similar performance for the external nitrogen atoms (*N6* and *N8*) compared to the central one (*N7*). The same analysis applied to **2** and **3** provides a different impact of water, particularly in **3**. The O-helix (including residues 656–672) is a single α-helix located within the fingers subdomain and the active site (see Figure 1). It is normally used to differentiate between closed and open conformations [76]. In our simulations, important changes indeed take place in the finger domain of the enzyme, mainly in the orientation of the O-helix near the primer terminus and the dNTP-binding site. In particular, the closeup view of the O-helix and its superimposition of the most representative structures of all the examined ternary complexes (Taq:DNA:dNTP) evidence a better similarity in the cases of **2** and **3** with dGTP compared to **1** (Figure 5).

This behavior is a significant consequence of the presence of water molecules in the cavity lodging the **1**, causing a deviation of the O-helix (see Figure 4a) by about 35°. The structures of Taq:DNA:**2** and Taq:DNA:**3** show a similar orientation of the O-helix, as indicated by the plot of distance during the simulation time between the centers of mass of the O-helix and of the dNTP, which are similar to each other (around 6–7 Å), and with dGTP, which reveals a more constant value between 6 and 7 Å. On the other hand, the ternary complex Taq:DNA:**1** moves away from this value, reaching 10 Å (Figure 4b).

**Figure 6:**
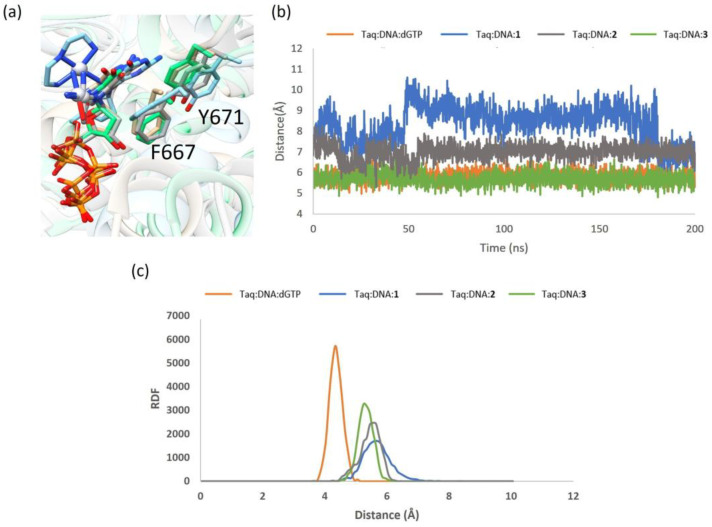
(**a**) Superposition of the four investigated systems, focusing on the different dNTP and F667 and Y671 amino acids. (**b**) Measured *N3*_dNTP_-OH_Y671_ and (**c**) calculated radial distribution function (RDF) for the center of mass of the guanine-_dNTP_-phenol ring-_Y671_ pair obtained for the four different systems, {where: **1** = [Pt(dien)(*N7*-dGTP)]; **2** = *cis*-[Pt(NH_3_)_2_Cl(*N7*-dGTP)]; **3** = *cis*-[Pt(NH_3_)_2_(H_2_O)(*N7*-dGTP)]}.

Another aspect strictly related to the O-helix behavior and corresponding to that of the two aromatic residues therein placed, Y671 and F667, was carefully monitored in all four simulations. By looking at Figure 6a, it is possible to note that, despite the similarity of the side chain with Y671, the F667 in the case of **1** (cyan line of the figure) exhibits a different orientation from the other dNTPs, pointing its aromatic ring parallel to that of **1** and establishing a stacking interaction. This behavior is once again a consequence of the different platinated moiety, which, due to its bulky and rigid nature, forces the nucleobase to orient in such a way as to interact with the closest aromatic amino acid (F667) that, along with Y671, forms a cleft where the nucleobase of the incoming dNTP is positioned.

A deeper analysis of the dynamic behavior of the residues belonging to the O-helix can provide insights on the dNTPs incorporation that are useful for properly discriminating between the three different platinated nucleotides. In Figure 6b, the dynamic behavior of tyrosine 671 in all the four systems investigated is reported. This residue is highly conserved throughout evolution in DNA polymerase family A from bacteria to humans and plays a crucial role in bypassing non-instructive lesions by DNA polymerases [77]; furthermore, it is implicated in establishing hydrogen bond networks with the incoming nucleotide residue.

Additional attention was given to this residue by analyzing the distance between its -OH moiety and the *N3* of the incoming dNTP as a putative indicator of the presence of a hydrogen bonding interaction in some cases [76,77]. For all the investigated ternary complexes, the distance values during the MD simulations revealed that no H-bond interactions took place since they were longer than 5 Å. In particular, for **1**, the highest value of this distance was observed. Taking into account the π stacking distance between the aromatic ring of Y761 and that of dNTPs (Figure 6c), Y671 is positioned in a way that it nicely stacks to the natural dGTP with a distance of 4 Å, as evidenced by a peak with a higher intensity than the longer distances and lower peaks in the case of the platinated nucleotides.

Another important amino acid residue monitored was K663, which is believed to act as a general acid during the catalysis of nucleotide bond formation and to facilitate the formation of canonical WC base pairing. The distance of its -NH_3_ moiety from the oxygen that bridges the α- and β-phosphate groups of the triphosphate moiety is 3–5 Å and shows a common trend for all four ternary complexes (Figure S4). In the most representative structure obtained by cluster analysis of each ternary complex, the ammonium moiety of lysine is perfectly oriented towards the bridged oxygen between the P_α_ and P_β_ atoms, indicating their coherent location around the Mg^2+^ ions in all four ternary complexes.

For all the considered species, the interaction occurring between Taq:DNA systems and the nucleotides was qualitatively analyzed in the mean of MMPBSA calculations, and, for all the ternary systems, highly favorable thermodynamics were obtained, thus highlighting the affinity of the Taq:DNA system to the substrates (see Table S2). In detail, the best affinity was identified for the natural substrate dGTP, as expected, while comparable values were obtained for species **1**–**3**, making the identification of a better affinity for the Pt-containing species difficult. Instead, a different energetic trend was observed, focusing on the energetic examination of the interactions established between the amino acids of the catalytic pocket and nucleotides. This was examined through calculations of per-residue decomposition energies, which provided an estimation of the energetic impact of the specific residues of the binding site implicated in the non-covalent interactions with the dNTPs. It emerged that, during the molecular dynamics simulations, more stabilizing protein-ligand interactions took place in the case of dGTP, species **3** and **4** (see Table S2 for details), which was qualitatively in agreement with the above-mentioned results.

It is known that Taq-DNA polymerase is capable of inducing the formation of a Watson–Crick-like pair in its incorporation site, even with an unnatural base [65]. Therefore, more attention was focused on the Watson–Crick geometry essential to the recognition of the “cognate” nucleotides in the active site of DNA polymerases [78,79,80].

From Figure S5, it is possible to see that **2** and **3** exhibit similar behavior to the natural dGTP, remaining perfectly paired for the duration of the simulation except for the most frequent deviations of HN- -O distance between **2** and **3**. This does not take place in the case of **1**, where the presence of the very bulky group is detrimental to its cytotoxic potency due to the loss of important stabilizing interactions.

The *C1′*–*C1′* distance represents another important structural parameter that is strictly related to the base pair. As widely reported in previous computational works [81,82,83,84], it also accurately accounts for the adopted geometry between the template and the incoming dNTP, in our case pyrimidine (dC) and purine (dGTP), respectively, indicating how the base pair fits well within the active site. The behavior of this distance during MD simulation can become a determining factor for the evaluation of differences among the platinated moiety-containing nucleotides (**1**, **2**, and **3**).

**Figure 7:**
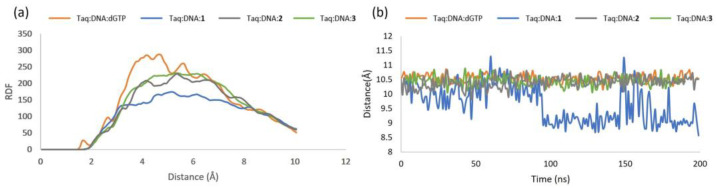
(**a**) RDF calculated for guanine-_dNTP_-cytosin-_dC551_ pair, and (**b**) *C1′*_dNTP_-*C1′*_dC551_ distance observed in the MD simulations of the four investigated systems, {where: **1** = [Pt(dien)(*N7*-dGTP)]; **2** = *cis*-[Pt(NH_3_)_2_Cl(*N7*-dGTP)]; **3** = *cis*-[Pt(NH_3_)_2_(H_2_O)(*N7*-dGTP)]}.

Observing Figure 7, the C1′–C1′ distance (10.5 ± 0.1 Å) for the dC:dGTP base pair assumes values closer to the ideal C1′–C1′ distance in the canonical G:C base pair model (10.8 Å) [84,85] while the same distance in the dC:**2** and dC:**3** base pairs was slightly shorter (10.4 ± 0.2 Å). A very peculiar trend is observed in the case of dC:**1**, whose *C1′*–*C1′* distance starting from 10 Å in the second half of the simulation time arrives at a shorter distance (9.2 ± 0.5 Å) than that observed in the canonical base pair. As confirmation of this, the trend of the hydrogen bond pattern in the base pair during the simulation time (Figure S5) emerges, showing that dC:dGTP, dC:**2**, and dC:**3** base pairs adopt a planar Watson–Crick (WC) geometry within the “duplex” DNA, except for some exceptions in the case of the HN-O distance for both dC:**2** and dC:**3** systems. However, dC:**1** shows a very dissimilar trend from canonical WC geometry for all three H-bonds monitored during the whole simulation since no base pair is evidenced (Figure S5).

**Figure 8:**
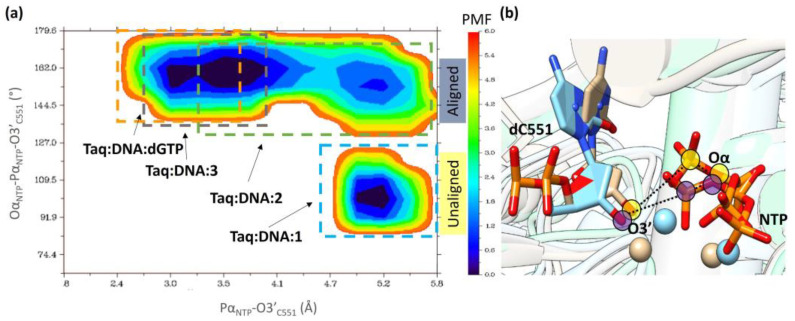
(**a**) Distribution of *O3′*_d551_−Pα_dNPT_−Oα_dNPT_ angle and *O3′*_d551_−Pα_dNPT_ distance obtained for the four investigated systems {where **1** = [Pt(dien)(*N7*-dGTP)]; **2** = *cis*-[Pt(NH_3_)_2_Cl(*N7*-dGTP)]; **3** = *cis*-[Pt(NH_3_)_2_(H_2_O)(*N7*-dGTP)]}, and (**b**) the atoms involved in the aligned/unaligned conformations.

A geometrical contribution to the subsequent chemical reaction, consisting of a nucleophilic attack from the primer base to the incoming nucleotide, arises from monitoring the *O3′*−Pα distance and the *O3′*−Pα−Oα angle, depicted in Figure 8. This was obtained by the scatter plot of the two geometric parameters, where correlations of each distance-angle value were analyzed. This allowed the identification of aligned and unaligned conformations as a function of the *O3′*−Pα−Oα angle (chosen cut-off angle = 140°, see Figure 8). It turned out that the *O3′* of the dC551 primer was optimally aligned for the nucleophilic attack of the Pα atom of the incoming dNTP, owing to the *O3′*−Pα distance (*O3′*−Pα−Oα angle): 3.3 ± 0.2 Å (163° ± 5) for dGTP, 5.3 ± 0.1 Å (106° ± 4) for **1**, 3.8 ± 0.2 Å (165° ± 5) for **2**, and 4.4 ± 0.7 Å (163° ± 6) for **3**. This finding suggests that dGTP, as well as **2** and **3**, did not flip out of the active site during the simulation, unlike **1** (Figure 8). This distance is a reaction coordinate implicated in dNTP incorporation, which occurs via the nucleophilic attack of the primer terminal 3′-OH on the α-phosphorus of the incoming dNTP, a reaction catalyzed by the Taq DNA polymerase. Despite the good value of the *O3′*−Pα distance for all the examined dNTPs, in the **1**-containing ternary complex, the *O3′*_dC551_ in the **1** system results unaligned with respect to other incoming dNTPs, as confirmed by the angle value of 106°, suggesting a very poor “reactive” orientation for its incorporation into the growing primer strand, with the consequent release of the pyrophosphate (PPi). This positioning can be linked to a less catalytic conformation and would require a higher amount of activation energy for the reaction to occur.

Umbrella sampling simulations [86] on the *O3′*_P:dC551_-P_α dNTP_ distance corroborate these outcomes (Figure S6). Since an energy penalty for the realignment is found, further calculations at the QM or QM/MM level are required to quantify the real activation barrier for this step, but this aspect is beyond the scope of the present study. Snapshots of each trajectory are finally reported in the Supplementary Material file (see Figures S7–S10), while the results on the replicas are shown in Figures S11 and S12.

### Implications for the Mechanism of Action of Cisplatin

It is well known that the major adducts formed by the interaction of cisplatin with DNA are *cis*-GG (≈47–50%) and *cis*-AG (≈23–28%) 1,2-intrastrand cross-links, involving adjacent nucleobases. In addition, *cis*-GNG 1,3-intrastrand cross-links are also formed (8–10%), together with interstrand cross-links and monofunctional adducts with a single guanine (2–3%) [87].

It is also known that free dPTP {5′-(2′-deoxy)-purine-triphosphate} normally occurs in the cell cytoplasm and nucleus at relevant concentrations [58,59,61,63,64,88]. Therefore, we assumed that *cis*-[Pt(NH_3_)_2_Cl(*N7*-dPTP)] and *cis*-[Pt(NH_3_)_2_(H_2_O)(*N7*-dPTP)] complexes could be formed in the cell cytoplasm by direct interaction of free dPTP with cisplatin. This aspect is even more clear when considering: (i) the higher steric accessibility and reactivity of free purine nucleobases compared to those strictly stacked into DNA; (ii) the higher concentration of free dNTPs generally found in cancer cells compared to healthy cells [89]; and (iii) the much higher sensitivity of cancer cells to cisplatin when at the border between G1 and S phases of the cell cycle, when intracellular concentration of free dNTPs is maximized [88,90,91,92].

Indeed, if we do not consider the eventual formation of Pt-dATP complexes due to the clear preference of cisplatin and platinum drugs for guanine residues, the previously mentioned factors taken together concur to enhance the direct reactions between cisplatin, its aquated species, and the free dGTP. This could occur directly in the cell cytoplasm and nucleus, leading to the formation of cisplatin-dGTP mono-adducts of the types *cis*-[Pt(NH_3_)_2_Cl(*N7*-dGTP)] (**2**) and *cis*-[Pt(NH_3_)_2_(H_2_O)(*N7*-dGTP)] (**3**), here considered. These are interesting biomimetic substrates whose role in the mechanism of action of cisplatin and their suitability to follow some of the metabolic pathways of endogenous nucleotides need to be clarified [41,93].

In this work, we studied at the atomistic level, by unbiased MD simulations, the incorporation mechanism into the model Taq DNA polymerase of previously considered [Pt(dien)(*N7*-dGTP)] (**1**) [58,59,61,63,64], in comparison with the elusive reactive complexes **2**–**3**. This approach permitted us to shed light on some key aspects of the role of direct interactions occurring between cisplatin and free dGTPs in its mechanism of action. We used complexes **1**–**3** as substrates for the Taq DNA polymerase, showing that complex **1** has an affinity with the DNA polymerase much lower than that of complex **2** and related aquo species **3**. Interestingly, it resulted in the behavior of **2**–**3** for the Taq DNA polymerase strictly resembling that of natural dGTP. Instead, complex **1** has some difficulties establishing geometrically productive interactions inside the active site of the polymerase. This is consistent with previous experimental findings demonstrating a much higher (15–20 times) rate of incorporation for the natural dGTP if compared to complex **1** [61,63]. On the contrary, complexes **2**–**3** can be easily recognized by Taq polymerase, producing Watson–Crick base pairings very similar to those occurring with the natural dGTP. This can be responsible for the lower rate of incorporation of complex **1** compared to the metabolites **2**–**3**.

## 3. Methods

### 3.1. Preparation of the Model

**Figure 9:**
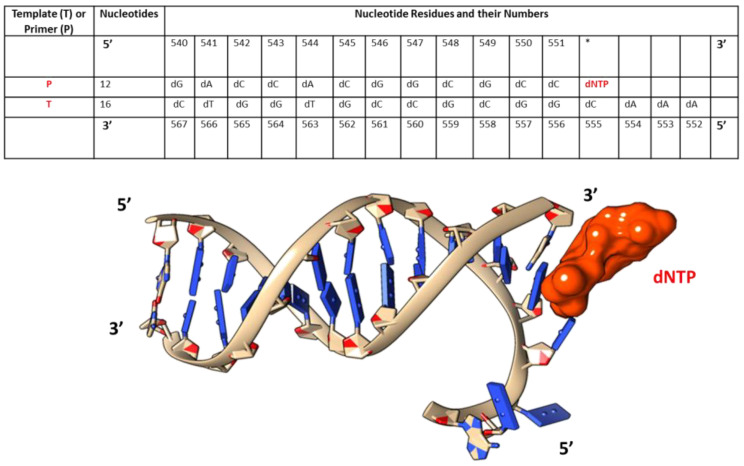
Schematic representation of the DNA-dNTP model adduct adopted in the present study. Residue numbers are reported following the PDB numeration [65]. Star (*) indicates the point of DNA replication process where the insertion of dNTP takes place.

The structures of all models of the dNTP-bound Taq DNA polymerase complexes are based on the single X-ray crystal structure containing 5-methylcytosine (5mC) (PDB 5YTD, resolution of 2.0 Å) [65]. The structure of the enzyme consists of 539 amino acid residues and includes two Mg^2+^ ions for the nucleotidyl transfer reaction, in addition to the oligonucleotide UGCCCGGG, which represents both the DNA primer and DNA template, as reported in detail in Figure 9. The guanine triphosphate already present in the structure was modified only on the base component to obtain the three modified nucleotides to the *N7* of dGTP by adding the platinated moiety, as depicted in Scheme 1 and represented in Figure 2.

The paired 5mC base was also modified to form its corresponding natural base (cytosine) using Gauss View [94]. 5mC is characterized by naturally occurring chemical modifications and is known as the fifth base in DNA. These modifications do not interfere with base pairing, allowing the formation of a good starting Watson–Crick geometry based almost exclusively on the available crystallographic data. Consequently, the four model systems are “accommodated” at the site of the polymerase surface corresponding to the insertion site where the recognition events take place, as can be seen in Figure 9.

### 3.2. MD Simulations

Before MD simulations of the metal-containing Taq systems, it was necessary to obtain parameters for all three platinated nucleotides (**1**–**3**). To do this, the Seminario Method was carried out as implemented in the MCPB module of the Amber16 software [95,96], starting from quantum mechanics calculations using the B3LYP functional coupled to the 6-31G* basis set [97,98]. Atomic charges were derived by fitting the electrostatic potential according to the Merz–Singh–Kollman scheme [99] using the RESP procedure. The Antechamber and Parmchk modules of Amber16 were employed to generate preparatory files to perform molecular mechanics (MM) relaxation of the complexes.

Three molecular dynamics simulations were performed to compare the behavior of *N7*-platinated guanines **1**–**3**, represented in Scheme 1, to the natural triphosphate nucleotide (dGTP). Each simulation lasted 200 ns and was carried out using the AMBER16 code [99], coupled with the FF14SB and FF99bsc0 force fields for the treatment of the protein [100] and the DNA [101,102], respectively. Each system was inserted in an orthorhombic box (96 × 78 × 84 Å^3^) with a buffer of 10 Å of TIP3P water molecules from the protein, and Na^+^ counter ions were added to neutralize the system (34 ions for Taq:DNA:dGTP, Taq:DNA:**1**, and Taq:DNA:**2** and 35 ions for Taq:DNA:**3**). An additional replica of 100 ns has been preliminarily carried out on each complex to detect any relevant conformational changes. A comparative analysis of the results in more detail is reported in the Supplementary Material file.

The solvated structures were first minimized by applying harmonic restraints on all atoms of the enzyme (50 kcal mol^−1^ Å^2^) using 5000 steps of the steepest descent algorithm, followed by 5000 steps of the conjugate gradient algorithm. In the second minimization step, the restraints on hydrogen atoms were released, with the third and fourth minimizations being conducted with and without protein backbone atom restraints, respectively. The same stepwise minimization procedure has been successfully applied to similar systems [81,103]. We then carried out a progressive heating phase from 0 to 310 K for 200 ps using the Langevin thermostat in the NVT ensemble, with a time step of 0.002 ps. The production phase was performed under the following conditions: integration step of the 2 fs coupling SHAKE algorithm; NPT ensemble at 1 bar pressure using the Berendsen barostat [104] with a time constant τp = 2.0 ps. The particle mesh Ewald summation method [105] was employed for the electrostatic potential long-range interactions with a 12 Å cut-off distance. RMSD-based clustering of the entire trajectories was performed according to the relaxed complex scheme (RCS) protocol implemented in Amber 16 to provide a sampled and energetically accessible conformational ensemble. After removing the overall rotations and translations by RMS fitting of the positions of the Cα atoms of the trajectory, the average binding clustering algorithm implemented in cpptraj [106] was applied to identify 10 clusters of representative conformations of the protein. The resulting MD trajectories were used to assess the magnitude of structural changes in terms of root mean square deviation (RMSD), propensity of a given residue or region to shift, and root mean square fluctuation (RMSF). The trajectories were saved every 0.1 ps and analyzed through the PTRAJ module.

### 3.3. MMPBSA

The binding free energies between the examined ligands (see Scheme 1) and Taq-DNA polymerase were calculated by solving the linearized Poisson–Boltzman equation using the Molecular Mechanics-Poisson–Boltzmann Surface Area (MM-PBSA) method, as implemented in Amber code 16 [99]. The igb flag value of five associated with a salt concentration of 0.1 M was used. The binding energies for the individual residues at 3 Å from the nucleotide triphosphate were decomposed to verify their individual contributions. For the calculations, 100 frames of each molecular dynamics (MD) trajectory over the last 100 ns were analyzed.

### 3.4. PCA

Principal component analysis (PCA) was performed using the cpptraj module of Ambertools 16 to extract the large-scale collective motions occurring in MD simulations of Taq:DNA:dGTP, Taq:DNA:**1**, Taq:DNA:**2**, and Taq:DNA:**3**. This procedure typically extracts information on the major conformational changes taking place along the MD trajectories.

### 3.5. PMF

Potential mean force (PMF) for the energy landscape based on the results of molecular dynamics simulations was obtained by MolAICal software [107,108], which allowed for the different behavior of the examined Pt-analogs inside the pocket of the Taq:DNA system to be evidenced.

### 3.6. Umbrella Sampling

In addition, considering the importance of the Pα_NTP_-O3′_DNA_ distance, umbrella sampling simulations were performed to restrain a reaction window corresponding to the transition slot from non-bonded-like to bonded-like conformations. The analysis of the potential of mean force (PMF) was carried out using the WHAM method [107,108]. The free energy difference is the driving force of the process of unbound/bound, which indicates the possible incorporation of a new base into the DNA strand.

## 4. Conclusions

In conclusion, we studied the binding mechanisms of the model platinated nucleotides [Pt(dien)(*N7*-dGTP)] (**1**), *cis*-[Pt(NH_3_)_2_Cl(*N7*-dGTP)] (**2**), and *cis*-[Pt(NH_3_)_2_(H_2_O)(*N7*-dGTP)] (**3**) to the Taq DNA polymerase using unbiased MD simulations. We differentiated the incoming platinated nucleotides during incorporation, observing that complex **1** had difficulty adopting the correct geometric interactions inside the active site. In contrast, the cisplatin metabolites **2**–**3** could be easily recognized, giving a Watson–Crick base pairing that strictly resembled that of natural dGTP. This corresponded to a higher energy barrier and lower rate of incorporation calculated for complex **1** compared to complexes **2**–**3**. This significant difference between complex **1** and complexes **2**–**3** suggests that the incorporation of complex **1**, observed in previous in vitro experiments, could be much less significant than that occurring with the cisplatin metabolites **2**–**3** [61,63], with relevant implications for the mechanism of action of cisplatin and analogue drugs.

These findings are important because they could lead to a better understanding of how to improve the design of new antitumor/antiviral drugs based on platinated nucleos(t)ides in order to optimize the interactions with DNA polymerases, favoring the appearance of desired pharmacological effects with modulation of the metabolic pathways of nucleotides. In this way, a parallel reduction of the side effects generally associated with the use of platinum drugs could also be pursued.

## Data Availability

Data are contained within the article or the Supplementary Material.

## Supplementary Materials

The supporting information can be downloaded at: https://www.mdpi.com/article/10.3390/ijms24129849/s1.
