# Supplementary material for: Incorporation of N7-Platinated Guanines into Thermus Aquaticus (Taq) DNA Polymerase: Atomistic Insights from Molecular Dynamics Simulations
Source: Int J Mol Sci. 2023 Jun 7;24(12):9849. doi: 10.3390/ijms24129849 (PMC10297917; doi:10.3390/ijms24129849)
Supplement: Supplementary file 1 [file ijms-24-09849-s001.zip › ijms-2398091-supplementary.pdf]

-Supplementary information-

Incorporation of *N7*-Platinated Guanine into Thermus Aquaticus DNA Polymerase (Taq DNA Polymerase): Atomistic Insights from Molecular Dynamics Simulations

Federica De Castro [1], Giada Ciardullo [2], Francesco Paolo Fanizzi [1], Mario Prejanò [2], Michele Benedetti [1,*] and Tiziana Marino [2,*]

[1] Dipartimento di Scienze e Tecnologie Biologiche ed Ambientali, Università del Salento, Prov.le Lecce-Monteroni, Centro Ecotekne, I-73100 Lecce, Italy

[2] Dipartimento di Chimica e Tecnologie Chimiche, Laboratorio PROMOCS cubo 14C, Università della Calabria, I-87036 RENDE (CS), Italy

*** Correspondence: michele.benedetti@unisalento.it; tiziana.marino65@unical.it**

Figure S1........ S2
Figure S2........ S4
Figure S3........ S5
Figure S4........ S5
Figure S5........ S6
Figure S6........ S7
Figure S7........ S7
Figure S8........ S8
Figure S9........ S9
Figure S10........ S10
Replicas of Taq:DNA:ligand complexes. S10
Figure S11........ S11
Figure S12........ S12
Table S1 .... S13
Table S2. .... S13
Parameters in Amber16 format. S14

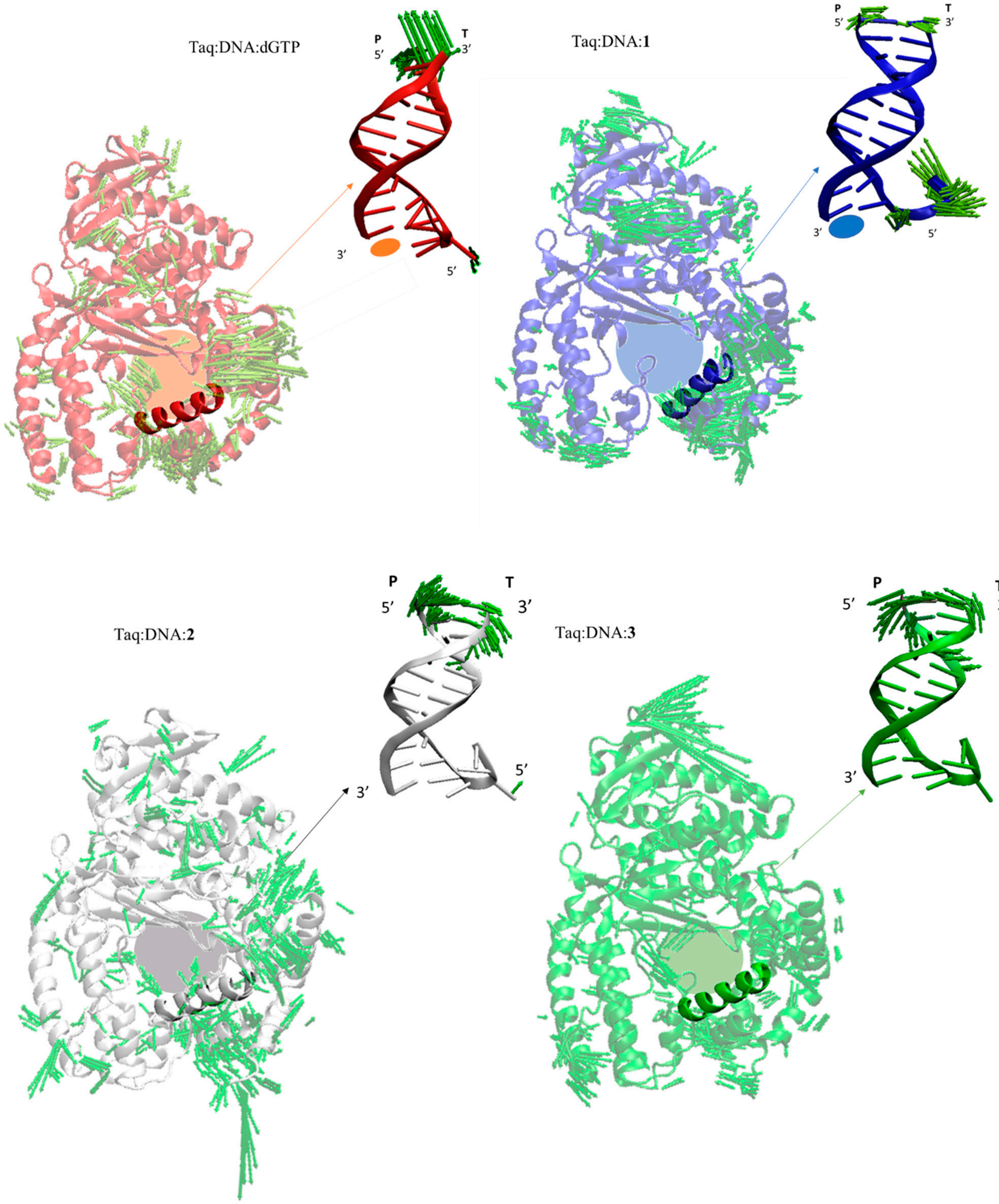

Figure S1. Principal component analysis (PCA) calculated for the investigated systems. Major fluctuations are represented as green arrows.

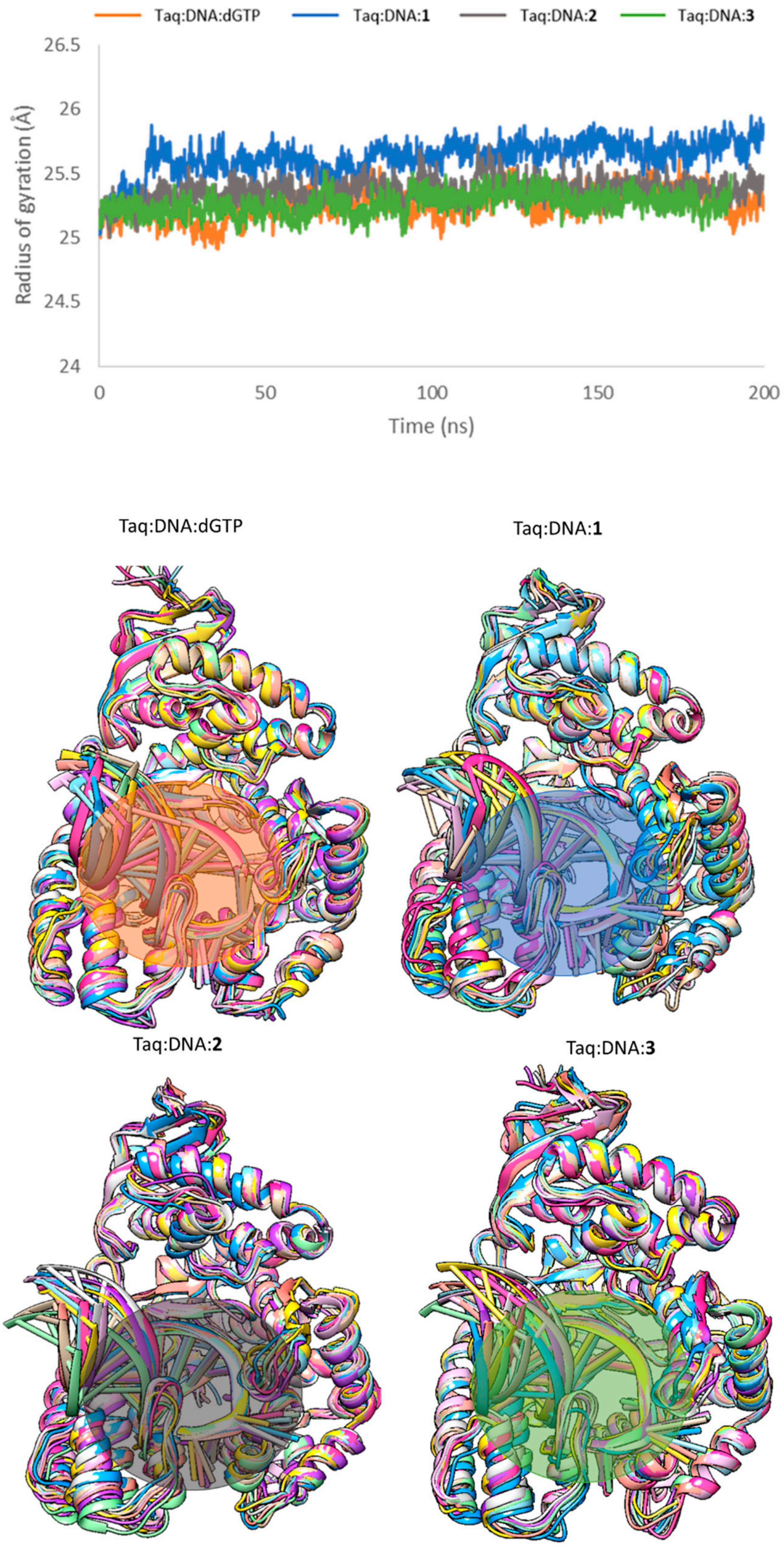

Figure S2. On the top, radius of gyration and, on the bottom, superposition of ten clustered geometries calculated for the investigated systems.

(a)

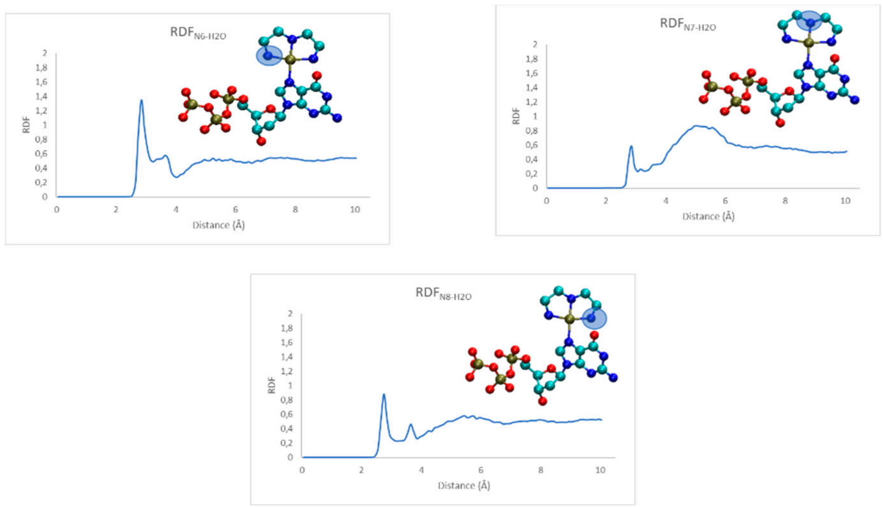

(b)

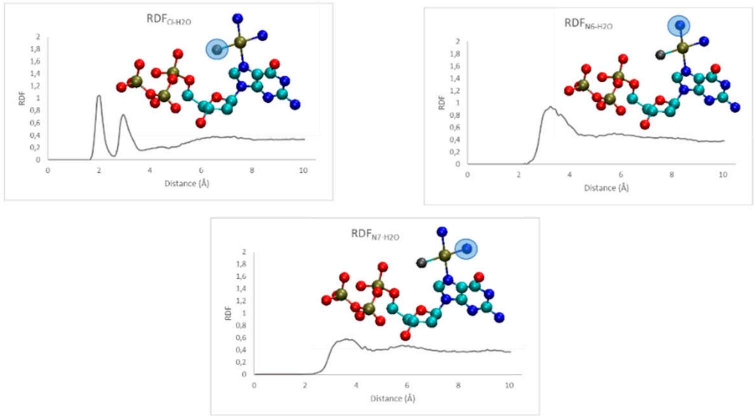

(c)

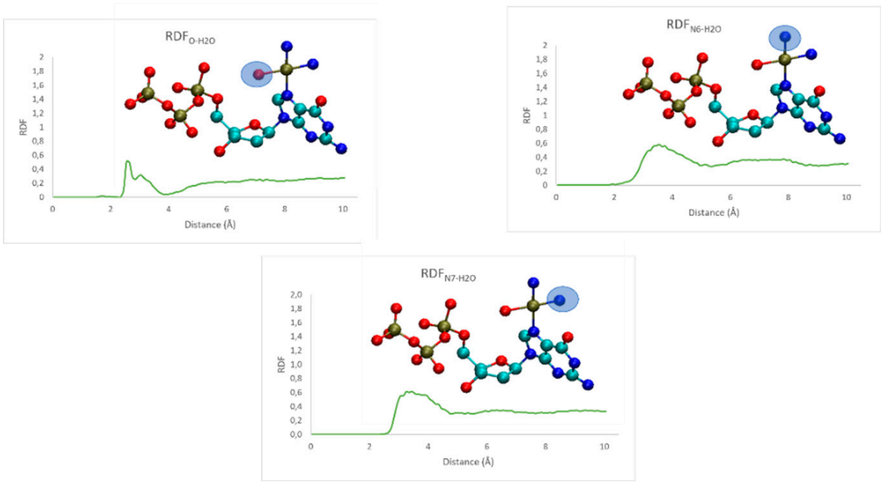

Figure S3. Radial distribution function (RDFs) of *N6*-H_2O, Cl-H_2O and *N7*-H_2O pairs, concerning the water molecule and the ligand to the Pt atoms (circled in the images), calculated for (a) Taq:DNA:**1**, (b) Taq:DNA:**2**, and (c)Taq:DNA:**3** complexes.

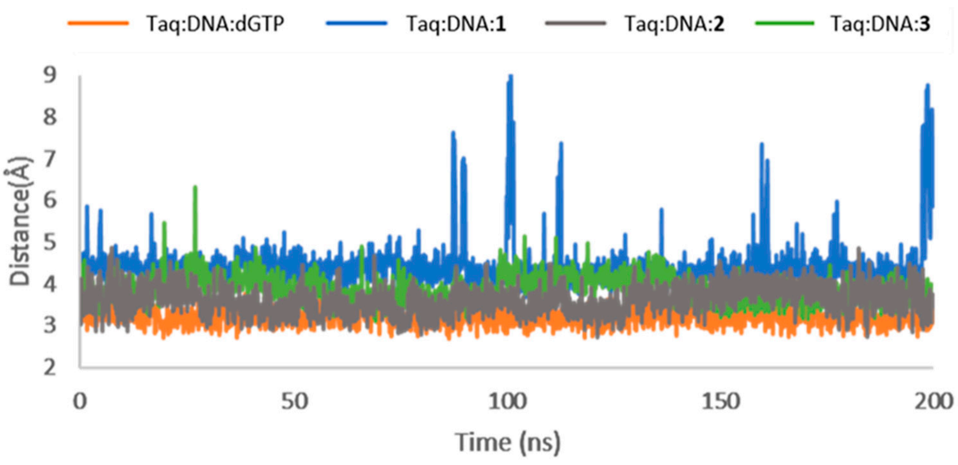

Figure S4. O_{dNTP}-$N\varepsilon_{K663}$ distance obtained from the molecular dynamics simulations of the four investigated systems.

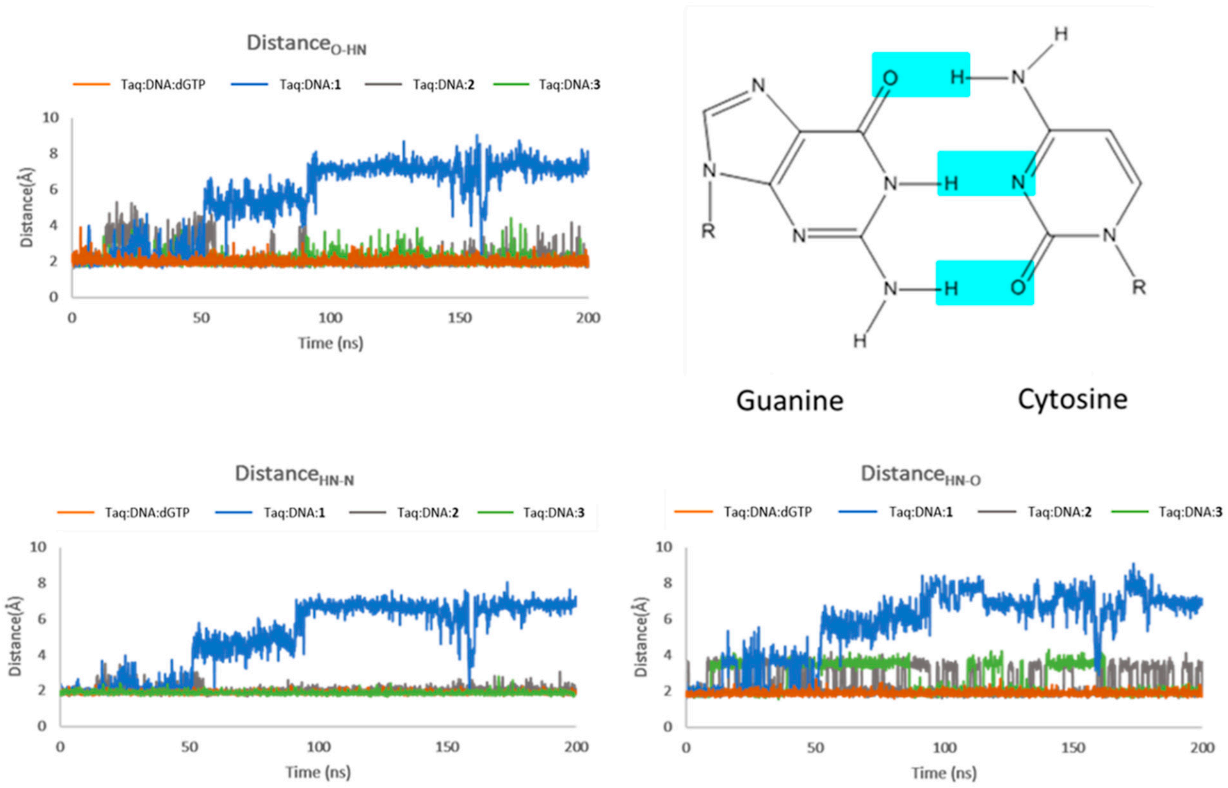

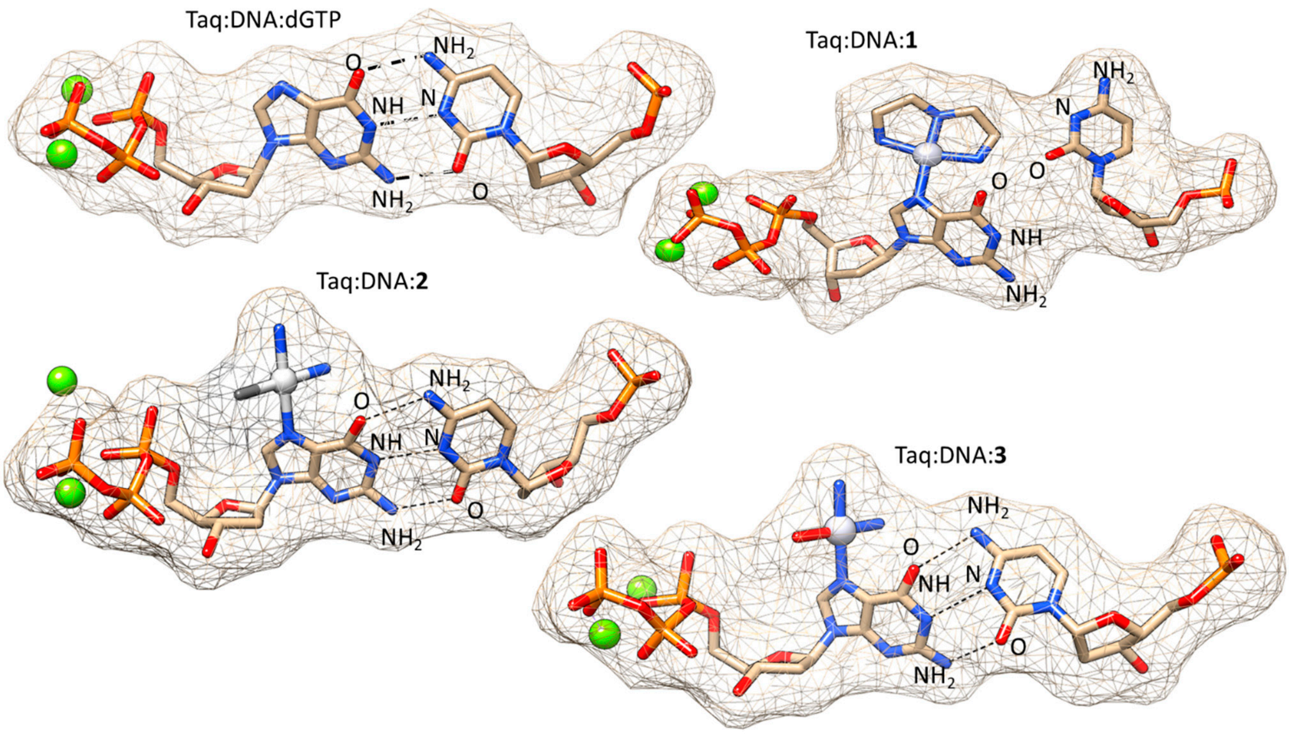

Figure S5. On the top, pairing of guanosine$_{dNTP}$-cytosine investigated in terms of hydrogen-bond parameters, obtained from the molecular dynamics simulations of the investigated ternary complexes. On the bottom, focus on the paired guanosine$_{dNTP}$-cytosine obtained from most representative clustered geometry.

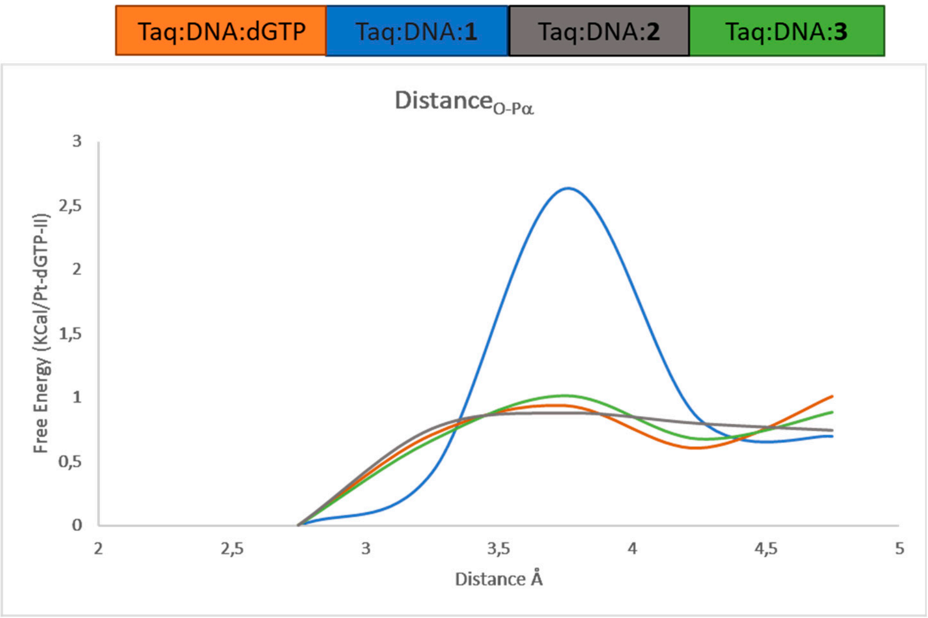

Figure S6. Potential of Mean Force (PMF) calculated for the four different ternary complexes, obtained from umbrella sampling simulations performed on the O$_{dC551}$-Pα$_{dNTP}$ distance.

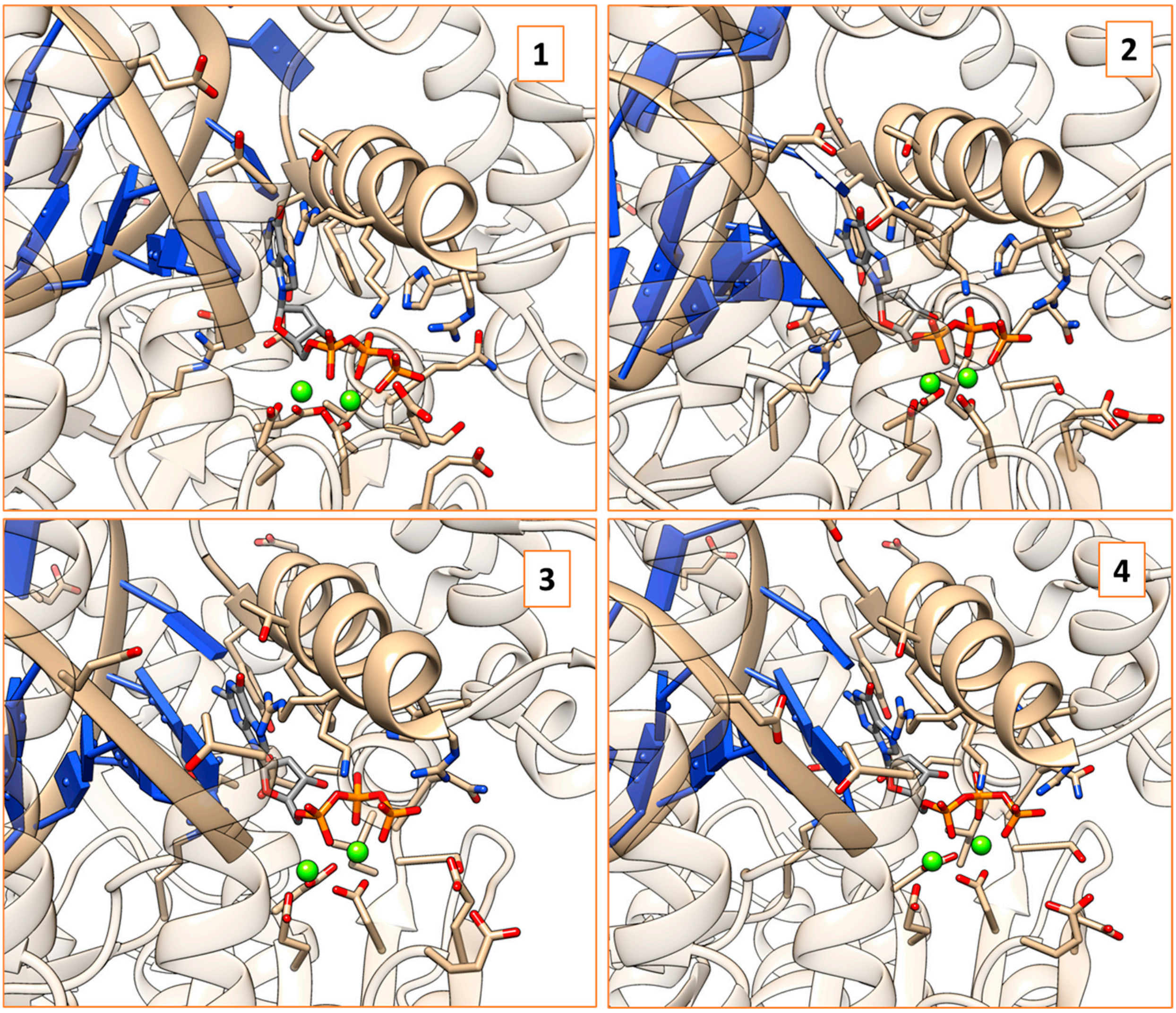

Figure S7. Snapshots of molecular dynamics trajectories, extrapolated at 50, 100, 150 and 200 ns, obtained for the Taq:DNA:dGTP system.

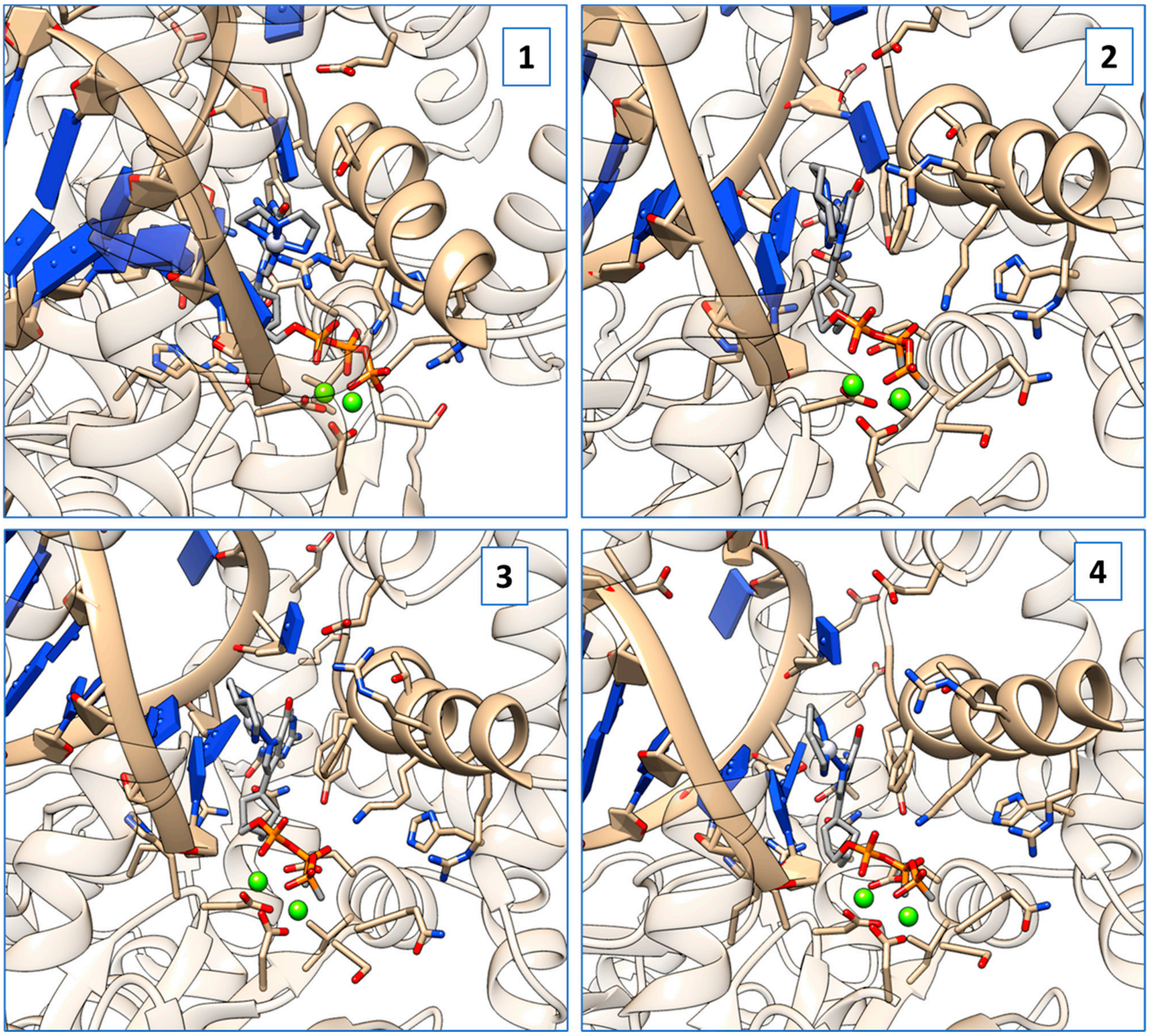

Figure S8. Snapshots of molecular dynamics trajectories, extrapolated at 50, 100, 150 and 200 ns, obtained for the Taq:DNA:**1** system.

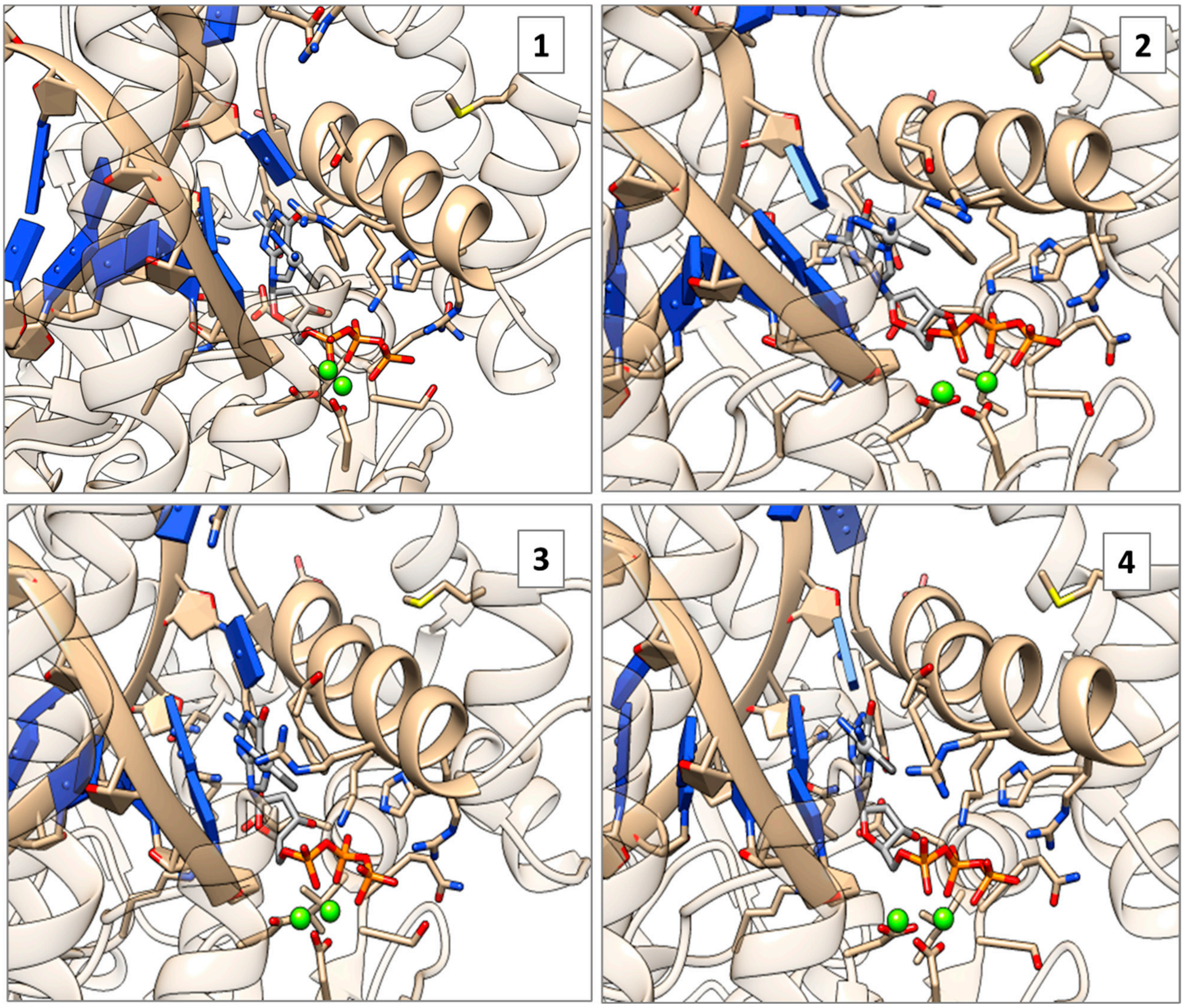

Figure S9. Snapshots of molecular dynamics trajectories, extrapolated at 50, 100, 150 and 200 ns, obtained for the Taq:DNA:**2** system.

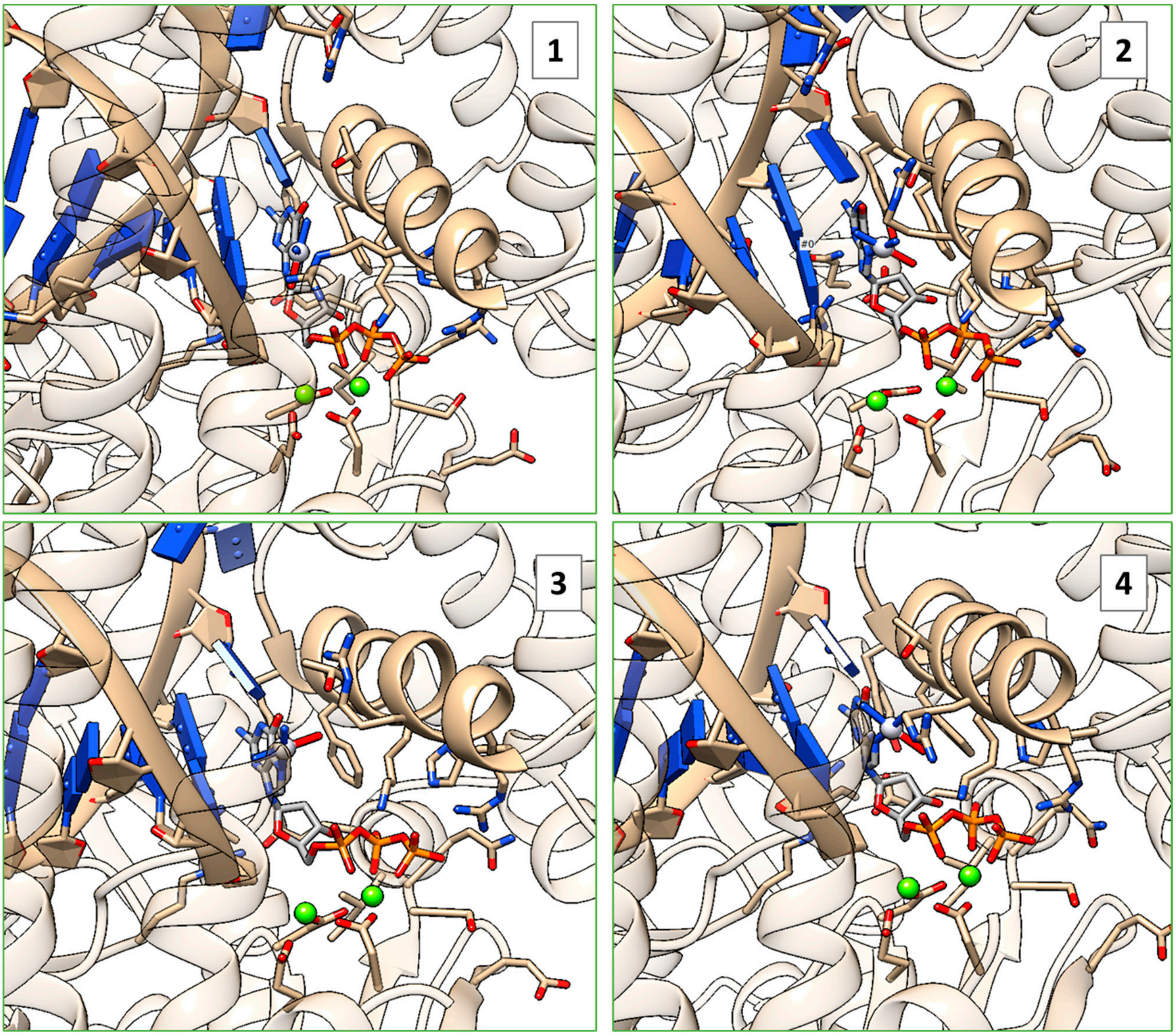

Figure S10. Snapshots of molecular dynamics trajectories, extrapolated at 50, 100, 150 and 200 ns, obtained for the Taq:DNA:**3** system.

Replicas of Taq:DNA:ligand complexes

A control on the reproducibility of the conformational behavior observed for all considered complexes was preliminary performed. In detail, additional 100 ns of molecular dynamics were carried out starting from input geometry obtained from the heating phase of the simulation and selecting the same procedure (conditions, algorithms, and software) discussed in the main text (see Methods section). At this stage of the investigation, the attention was mainly focused on the structural parameters considered in the initial part of the study, like RMSD, RMSF, radius of gyration and visual inspection of representative structures obtained from the hierarchical clustering procedure.

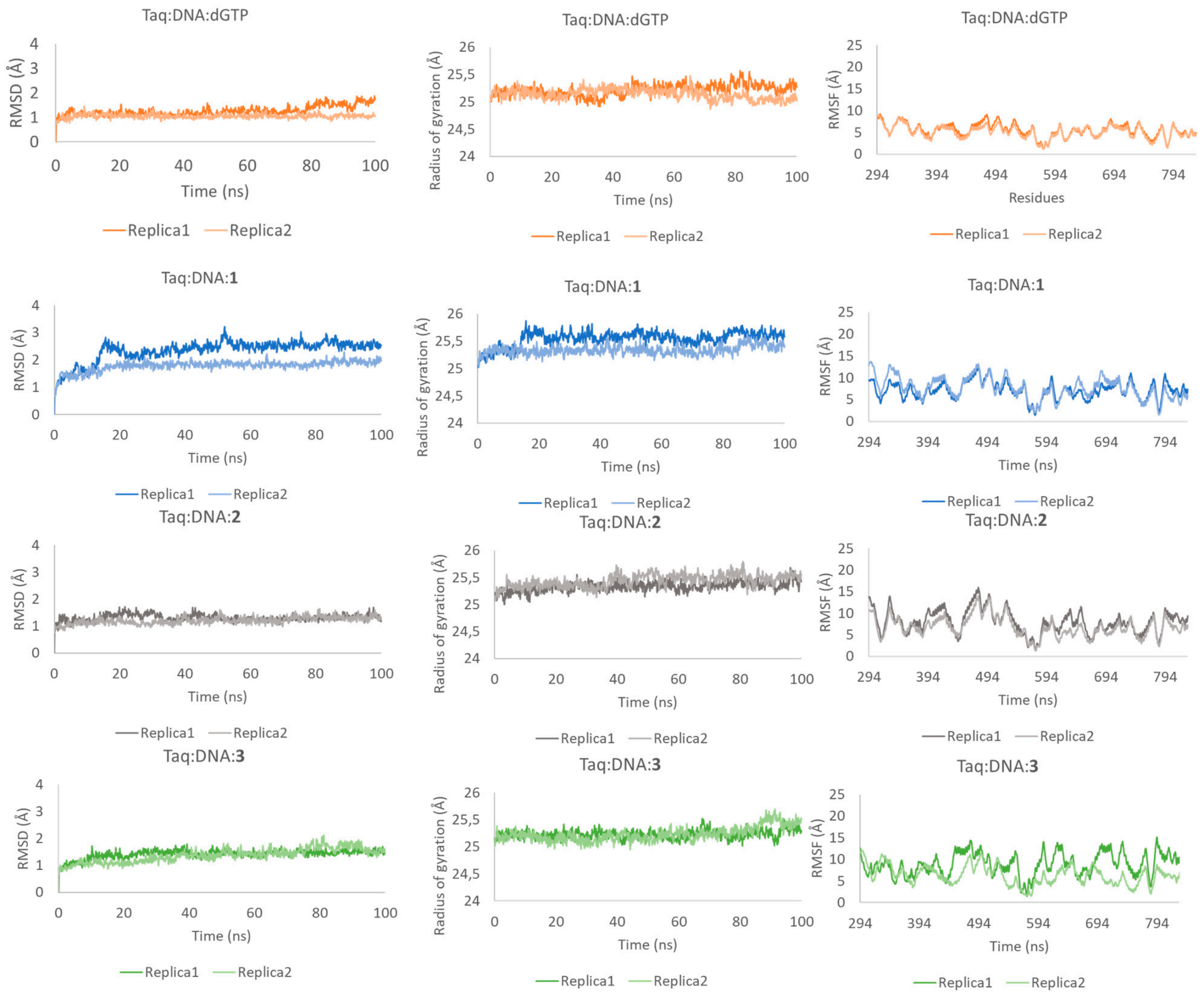

Figure S11. Structural parameters, considered for the analysis of the replicas carried out for the considered systems.

The comparison of each replica at 100 ns showed a reproducible RMSD trend for all the considered complexes, with higher values (>2 Å) obtained for Taq:DNA:**1**, as reported in **Figure S11**. Overall, the structural reproducibility was evinced by the analysis of RMSF and radius of gyration trends. In the case of the former, a good and superimposable trend between the replicas was obtained for Taq:DNA:dGTP and Taq:DNA:**1** in particular, in proximity of residues of the O-helix deputed to the recognition of the ligands (from residue 656 to residue 672, see **Figure S11**). Also the superposition of the most populated cluster geometries (see **Figure S12**) further did not highlight any relevant structural variation of the secondary structures.

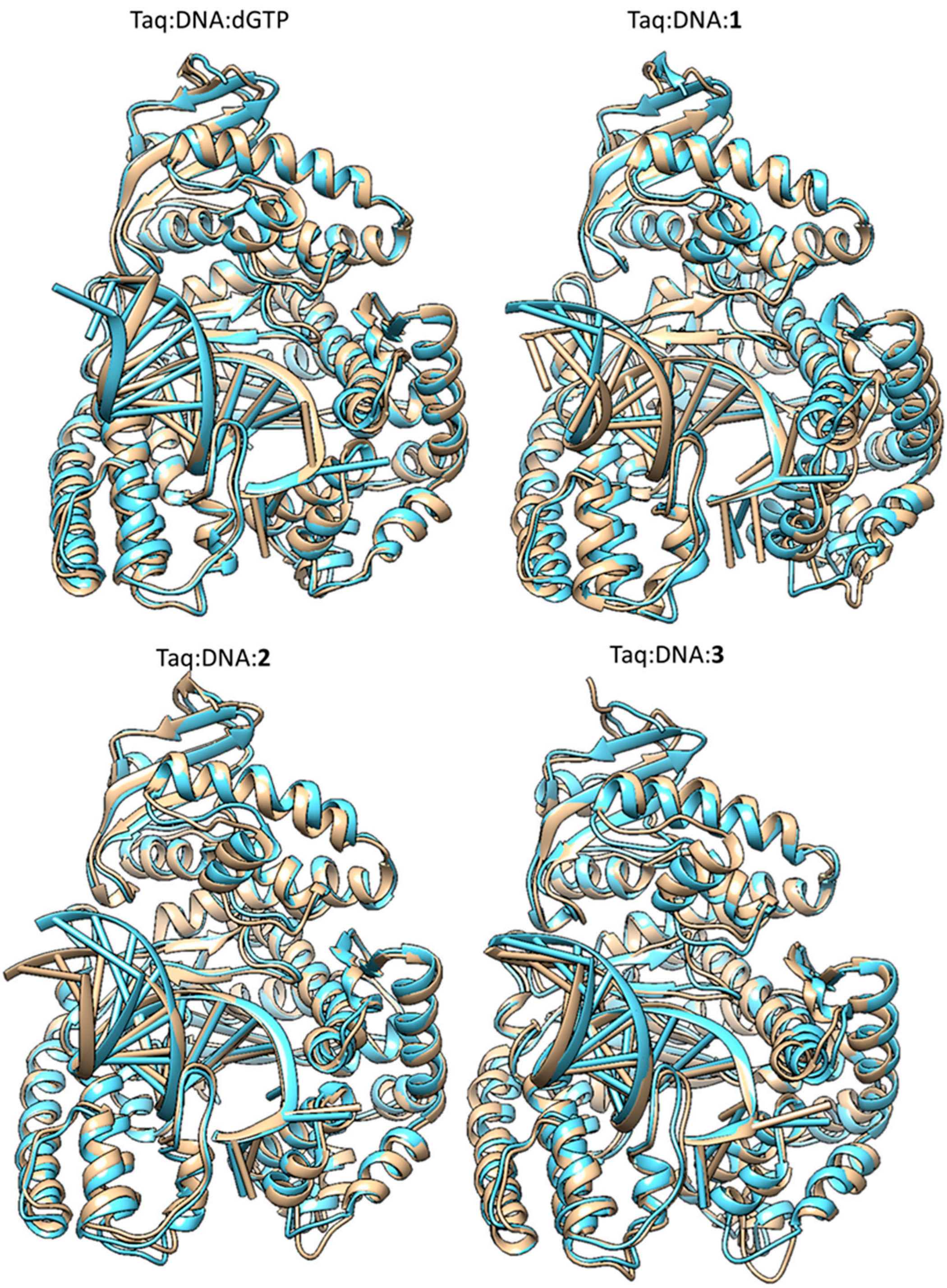

Figure S12. Superimposition of the most representative geometries obtained from hierarchical clustering procedure of replicas of the considered systems.

Table S1. Details of the ten clustered structures obtained form 200 ns of molecular dynamics simulation carried out for the investigated systems.

Taq:DNA:dGTP

Cluster	Frames	Frac	AvgDist	Stdev	Centroid	AvgCDist
0	7429	0.371	1.126	0.147	3491	1.57
1	5397	0.27	1.124	0.153	16140	1.539
2	2502	0.125	1.077	0.157	18983	1.478
3	1499	0.075	1.059	0.129	8313	1.436
4	1405	0.07	1.045	0.135	10719	1.472
5	1086	0.054	1.063	0.166	11386	1.482
6	365	0.018	1.045	0.163	9408	1.503
7	181	0.009	0.82	0.101	5221	1.692
8	116	0.006	0.922	0.063	7834	1.545
9	20	0.001	0	0	12954	1.784

Taq:DNA:**1**

Cluster	Frames	Frac	AvgDist	Stdev	Centroid	AvgCDist
0	9164	0.458	1.187	0.155	15067	1.786
1	5018	0.251	1.164	0.161	7874	1.677
2	2869	0.143	1.188	0.169	2943	1.633
3	1088	0.054	1.122	0.148	364	1.831
4	706	0.035	1.095	0.172	1747	1.65
5	454	0.023	1.01	0.132	5336	1.815
6	334	0.017	1.02	0.225	10851	2.028
7	180	0.009	1.015	0.143	1320	1.826
8	124	0.006	0.973	0.104	46	2.283
9	63	0.003	0.965	0.258	2085	1.766

Taq:DNA:**2**

Cluster	Frames	Frac	AvgDist	Stdev	Centroid	AvgCDist
0	13410	0.67	1.087	0.127	17621	1.315
1	3968	0.198	1.124	0.139	3198	1.346
2	1268	0.063	1.043	0.128	441	1.504
3	609	0.03	0.924	0.103	11763	1.5
4	283	0.014	0.956	0.154	17076	1.426
5	207	0.01	0.971	0.147	6090	1.462
6	153	0.008	0.901	0.129	4309	1.442
7	46	0.002	1.032	0.173	8834	1.431
8	41	0.002	0.83	0.033	15191	1.499
9	15	0.001	0	0	4749	1.485

Taq:DNA:**3**

Cluster	Frames	Frac	AvgDist	Stdev	Centroid	AvgCDist
0	6143	0.323	1.067	0.131	8386	1.293
1	5327	0.28	1.019	0.109	15418	1.295
2	4185	0.22	1.066	0.119	3451	1.313
3	1292	0.068	1.011	0.12	1623	1.279
4	897	0.047	0.972	0.127	478	1.35
5	867	0.046	1.023	0.153	9172	1.38
6	193	0.01	0.977	0.047	11185	1.434
7	54	0.003	0.829	0.088	897	1.355
8	29	0.002	0	0	16953	1.599
9	14	0.001	0	0	8580	1.398

Table S2. Results of MMPBSA calculation and, in the next page, Per-residue decomposition analysis carried out for all investigated systems.

Taq:DNA:dGTP

Energy Component	Average (kcal/mol)	Std. Dev.	Std. Err. Of Mean
VDWAALS	-30,5895	6,4703	0,647
EEL	50,7178	72,0463	7,2046
EPB	-217,0776	59,6789	5,9679
ENPOLAR	-3,8233	0,0552	0,0055
EDISPER	0	0	0
D G gas	20,1766	70,3387	7,0339
D G solv	-220,9009	59,6974	5,9697
**D TOTAL**	**-200,7243**	**14,1667**	**1,4167**

Taq:DNA:**1**

Energy Component	Average (kcal/mol)	Std. Dev.	Std. Err. Of Mean
VDWAALS	-16,8036	6,8919	0,6892
EEL	-464,87	58,0147	5,8015
EPB	376,4979	49,8608	4,9861
ENPOLAR	-5,64	0,1595	0,016
EDISPER	0	0	0
D G gas	-479,1343	58,2689	5,8269
D G solv	370,8578	49,846	4,9846
**D TOTAL**	**-108,2765**	**24,2667**	**2,4267**

Taq:DNA:**2**

Energy Component	Average (kcal/mol)	Std. Dev.	Std. Err. Of Mean
VDWAALS	-41,0381	5,8275	0,5828
EEL	141,7018	43,4112	4,3411
EPB	-202,5309	38,0495	3,8049
ENPOLAR	-4,8065	0,0971	0,0097
EDISPER	0	0	0
D G gas	100,6637	42,079	4,2079
D G solv	-207,3374	38,0494	3,8049
**D TOTAL**	**-106,6737**	**9,0736**	**0,9074**

Taq:DNA:**3**

Energy Component	Average (kcal/mol)	Std. Dev.	Std. Err. Of Mean
VDWAALS	-40,2914	7,2343	0,7234
EEL	-79,3406	31,1848	3,1185
EPB	29,1732	29,7848	2,9785
ENPOLAR	-4,8069	0,121	0,0121
EDISPER	0	0	0
D G gas	-119,632	31,2792	3,1279
D G solv	24,3662	29,7683	2,9768
**D TOTAL**	**-95,2657**	**9,3905**	**0,939**

Taq:DNA:dGTP

Resid Number	ΔG internal (kcal/mol)	ΔG vdw (kcal/mol)	ΔG eel (kcal/mol)	ΔG pol (kcal/mol)	ΔG TOT (kcal/mol)
R573	0	-0,482	-62,407	57,618	-5,271
D610	0	-0,951	145,217	-132,452	11,814
Y611	0	-0,722	19,885	-14,951	4,212
S612	0	-1,199	-14	11,632	-3,567
N613	0	-1,933	-19,824	14,504	-7,253
I614	0	-1,529	-8,493	6,414	-3,608
E615	0	-1,504	50,798	-41,045	8,249
H639	0	-1,498	-19,261	14,951	-5,808
R659	0	0,151	-127,343	108,912	-18,28
R660	0	-0,108	-81,505	73,89	-7,723
K663	0	-1,006	-158,545	140,663	-18,888
F667	0	-3,143	-5,433	5,047	-3,529
Y671	0	-0,756	-1,533	0,804	-1,485
Q754	0	-0,669	-0,542	-0,223	-1,434
D785	0	0,593	147,559	-128,492	19,66

Taq:DNA:**1**

Resid Number	ΔG internal (kcal/mol)	ΔG vdw (kcal/mol)	ΔG eel (kcal/mol)	ΔG pol (kcal/mol)	ΔG TOT (kcal/mol)
R573	0	-0,723	-35,935	31,866	-4,069
D610	0	0,402	127,891	-117,256	10,635
E615	0	0,236	44,809	-36,41	8,399
K663	0	-0,993	-81,605	81,77	0,165
F667	0	-3,886	-1,435	2,759	1,324
Y671	0	-0,817	-1,536	1,373	-0,163
N750	0	-0,271	0,445	-0,865	-0,42
Q754	0	-1,316	-3,169	1,478	-1,691
H784	0	-0,977	-0,325	1,241	0,916
D785	0	-1,206	127,557	-113,404	14,153

Taq:DNA:**2**

Resid Number	ΔG internal (kcal/mol)	ΔG vdw (kcal/mol)	ΔG eel (kcal/mol)	ΔG pol (kcal/mol)	ΔG TOT (kcal/mol)
R573	0	-1,034	-50,563	49,916	-1,681
D610	0	-0,988	112,165	-106,401	4,776
Y611	0	-0,423	15,648	-12,508	2,717
S612	0	-1,4	-12,007	11,01	-2,397
N613	0	-0,996	-14,656	13,088	-2,564
I614	0	-1,939	-5,967	5,158	-2,748
E615	0	-1,708	43,88	-37,804	4,368
H639	0	-1,4	-15,216	12,318	-4,298
R659	0	-0,036	-97,172	86,508	-10,7
R660	0	-0,213	-47,285	45,826	-1,672
K663	0	-0,992	-115,588	107,621	-8,959
T664	0	-0,615	0,281	1,179	0,845
F667	0	-3,712	-4,612	4,288	-4,036
Y671	0	-0,912	-0,288	0,158	-1,042
Q754	0	-0,728	-2,868	0,479	-3,117
D785	0	-1,045	105,871	-97,417	7,409

Taq:DNA:**3**

Resid Number	ΔG internal (kcal/mol)	ΔG vdw (kcal/mol)	ΔG eel (kcal/mol)	ΔG pol (kcal/mol)	ΔG TOT (kcal/mol)
R573	0	-0,579	-31,388	27,441	-4,526
D610	0	-0,599	93,879	-91,475	1,805
Y611	0	-0,844	12,952	-11,749	0,359
S612	0	-1,128	-10,15	9,32	-1,958
N613	0	-2,065	-12,516	11,309	-3,272
I614	0	-2,001	-6,058	5,243	-2,816
E615	0	-2,437	28,489	-18,935	7,117
H639	0	-1,208	-11,017	10,735	-1,49
R659	0	-0,05	-69,949	63,986	-6,013
R660	0	-0,368	-30,894	30,703	-0,559
K663	0	-0,075	-94,831	84,106	-10,8
F667	0	-3,815	-3,196	2,591	-4,42
Y671	0	-0,825	-0,666	0,181	-1,31
Q754	0	-1,048	-0,768	-0,363	-2,179
D785	0	-0,164	89,443	-86,925	2,354

Parameters in Amber16 format

Parameters complex 1

File mol2:

@<TRIPOS>MOLECULE

MOL

64 64 1 0 0

SMALL

bcc

@<TRIPOS>ATOM

1 P1 20.9120 7.7260 1.3950 p5 1
MOL 1.460900

2 O1 20.0490 8.6620 2.2080 o 1
MOL -0.960167

3 O2 22.0670 8.4040 0.6850 o 1
MOL -0.960167

4 O3 20.1600 6.7270 0.5290 o 1
MOL -0.960167

5 O4 21.4390 6.7790 2.5730 os 1
MOL -0.814800

6 P2 22.9680 6.3840 2.8980 p5 1
MOL 1.716800

7 O5 22.8700 5.1690 3.7780 o 1
MOL -0.944500

8 O6 23.8440 6.3660 1.7030 o 1
MOL -0.944500

9 O7 23.3530 7.5350 3.9730 os 1
MOL -0.817800

10 P3 24.3560 8.7590 3.6350 p5 1
MOL 1.611800

11 O8 23.8860 9.8320 4.5720 o 1
MOL -0.926500

12 O9 24.4740 8.9970 2.1750 o 1
MOL -0.926500

13 O10 25.7280 8.1780 4.2120 os 1
MOL -0.603200

14 C1 26.5990 7.3450 3.4680 c3 1
MOL 0.194400

15 C2 27.1220 6.2410 4.3650 c3 1
MOL 0.093100

16 O11 27.8070 6.7860 5.5060 os 1
MOL -0.453600

17 C3 26.0340 5.3420 4.9500 c3 1
MOL 0.148100

18 O12 25.6780 4.2710 4.0760 oh 1
MOL -0.686800

19 C4 26.7350 4.8090 6.1630 c3 1
MOL -0.113400

20 C5 27.6250 5.9470 6.6250 c3 1
MOL 0.250800

21 N1 26.8590 6.5940 7.7210 na 1
MOL -0.167900

22 C6 26.0230 7.6410 7.6390 cc 1
MOL 0.482400

23 N2 25.4980 7.9260 8.8270 Y1 1
MOL -0.647000

24 C7 25.9650 7.0220 9.6940 cd 1
MOL 0.212800

25 C8 25.8140 6.7290 11.1130 c 1
MOL 0.641000

26 O13 25.0580 7.4180 11.8400 o 1
MOL -0.667500

27 N3 26.5120 5.6870 11.6080 n 1
MOL -0.491400

28 C9 27.3280 4.9180 10.8510 cd 1
MOL 0.545800

29 N4 27.9970 3.8790 11.4040 nh 1
MOL -0.862800

30 N5 27.5110 5.1290 9.5380 nc 1
MOL -0.596100

31 C10 26.8720 6.1500 8.9400 cc 1
MOL 0.016800

32 C11 23.9650 9.4640 11.6690 c3 1
MOL 0.144300

33 C12 24.0130 11.4100 7.2970 c3 1
MOL 0.144300

34 N6 25.1670 10.9030 7.9230 Y2 1
MOL -0.931800

35 N7 23.6430 8.2890 10.9800 Y3 1
MOL -0.931800

36 C13 23.0130 10.4220 11.1140 c3 1
MOL 0.171300

37 C14 23.2640 11.5480 8.4720 c3 1
MOL 0.171300

38 N8 22.8980 10.8800 9.6470 Y4 1
MOL -0.861200

39 H1 27.4350 7.9350 3.0920 h1 1
MOL 0.028200

40 H2 26.0560 6.9070 2.6300 h1 1
MOL 0.028200

41 H3 27.8190 5.6260 3.7960 h1 1
MOL 0.061700

42 H4 25.1560 5.9240 5.2310 h1 1
MOL 0.180700

43 H5 27.3320 3.9330 5.9080 hc 1
MOL 0.064700

44 H6 26.0100 4.5590 6.9380 hc 1
MOL 0.064700

45 H7 28.5810 5.5700 6.9880 h2 1 MOL 0.052700

46 H8 25.8060 8.1800 6.7290 h5 1 MOL 0.172100

47 H9 26.4210 5.4700 12.5900 hn 1 MOL 0.313500

48 H10 24.9930 9.7690 11.4700 h1 1 MOL 0.034950

49 H11 23.8020 9.3380 12.7390 h1 1 MOL 0.034950

50 H12 24.1960 12.3540 6.8270 h1 1 MOL 0.034950

51 H13 23.1760 11.3390 11.6810 h1 1 MOL -0.005050

52 H14 22.0230 10.0640 11.3970 h1 1 MOL -0.005050

53 H15 22.3050 11.8120 8.0770 h1 1 MOL -0.005050

54 H16 22.0440 10.4230 9.3600 hn 1 MOL 0.454800

55 H17 28.6020 3.3070 10.8330 hn 1 MOL 0.366800

56 H18 27.8910 3.6760 12.3880 hn 1 MOL 0.366800

57 H19 22.9190 7.7940 11.4800 hn 1 MOL 0.372800

58 H20 23.3140 8.5210 10.0540 hn 1 MOL 0.372800

59 H21 24.7370 4.3100 3.8880 ho 1 MOL 0.493000

60 H22 25.9540 11.0230 7.3180 hn 1 MOL 0.372800

61 H23 23.6650 12.4580 8.8670 h1 1 MOL -0.005050

62 H24 23.5770 10.6990 6.6270 h1 1 MOL 0.034950

63 H25 25.3260 11.3960 8.7780 hn 1 MOL 0.372800

64 PT 24.2490 9.4200 9.2380 M1 1 MOL 2.000000

@<TRIPOS>BOND

1 1 2 1

2 1 3 1

3 1 4 1

4 1 5 1

5 5 6 1

6 6 7 1

7 6 8 1

8 6 9 1

9 9 10 1

10 10 11 1

11 10 12 1

12 10 13 1

13 13 14 1

14 14 15 1

15 14 39 1

16 14 40 1

17 15 16 1

18 15 17 1

19 15 41 1

20 16 20 1

21 17 18 1

22 17 19 1

23 17 42 1

24 18 59 1

25 19 20 1

26 19 43 1

27 19 44 1

28 20 21 1

29 20 45 1

30 21 22 1

31 21 31 1

32 22 23 2

33 22 46 1

34 23 24 1

35 24 25 1

36 24 31 2

37 25 26 2

38 25 27 1

39 27 28 1

40 27 47 1

41 28 29 1

42 28 30 2

43 29 55 1

44 29 56 1

45 30 31 1

46 32 35 1

47 32 36 1

48 32 48 1

49 32 49 1

50 33 34 1

51 33 37 1

52 33 50 1

53 33 62 1

54 34 60 1

55 34 63 1

56 35 57 1

57 35 58 1

58 36 38 1

59 36 51 1

60 36 52 1

61 37 38 1

62 37 53 1

63 37 61 1

64 38 54 1

@<TRIPOS>SUBSTRUCTURE

1 MOL 1 TEMP 0 **** **** 0 ROOT

File frcmod:

remark goes here

MASS

p5 30.970 1.538

o 16.000 0.434

os 16.000 0.465

c3 12.010 0.878

oh 16.000 0.465

na 14.010 0.530

cc 12.010 0.360

cd 12.010 0.360

c 12.010 0.616

n 14.010 0.530

nh 14.010 0.530

nc 14.010 0.530

h1 1.008 0.135

hc 1.008 0.135

h2 1.008 0.135

h5 1.008 0.135

hn 1.008 0.161

ho 1.008 0.135

M1 195.08

Y1 14.01 0.530 Sp2 N in non-pure aromatic systems, identical to nc

Y2 14.01 0.530 Sp3 N with three connected atoms

Y3 14.01 0.530 Sp3 N with three connected atoms

Y4 14.01 0.530 Sp3 N with three connected atoms

BOND

p5-o 529.55 1.487

p5-os 346.25 1.615

os-c3 284.76 1.432

c3-c3 232.52 1.538

c3-h1 375.92 1.097

c3-oh 293.40 1.423

oh-ho 563.51 0.973

c3-hc 375.92 1.097

c3-na 262.85 1.463

c3-h2 377.33 1.096

na-cc 354.49 1.380

cc-h5 403.49 1.082

cd-c 295.35 1.468

cd-cc 416.13 1.373

c -o 652.57 1.218

c -n 356.21 1.379

n -cd 353.83 1.381

n -hn 527.31 1.013

cd-nh 363.47 1.373

cd-nc 450.71 1.317

nh-hn 529.46 1.012

nc-cc 369.10 1.369

Y1-M1 80.2 2.1471 Created by Seminario method using MCPB.py

na-M1 80.2 2.1471 Created by Seminario method using MCPB.py

o-M1 80.2 2.1471 Created by Seminario method using MCPB.py

Y2-M1 107.7 2.1129 Created by Seminario method using MCPB.py

os-M1 80.2 2.1471 Created by Seminario method using MCPB.py

Y3-M1 120.0 2.0740 Created by Seminario method using MCPB.py

Y4-M1 166.0 2.0051 Created by Seminario method using MCPB.py

Y1-cd 441.1 1.3694 SOURCE1_SOURCE5 2269 0.0086

Y2-hn 392.4 1.0190 SOURCE3_SOURCE5
5944 0.0012

Y3-hn 392.4 1.0190 SOURCE3_SOURCE5
5944 0.0012

Y4-hn 392.4 1.0190 SOURCE3_SOURCE5
5944 0.0012

c3-Y2 325.9 1.4647 SOURCE1_SOURCE5
15206 0.0039

c3-Y3 325.9 1.4647 SOURCE1_SOURCE5
15206 0.0039

c3-Y4 325.9 1.4647 SOURCE1_SOURCE5
15206 0.0039

cc-Y1 525.4 1.3172 SOURCE3_SOURCE5
4612 0.0083

c3-Y1 525.4 1.3172 SOURCE3_SOURCE5
4612 0.0083

ANGLE

p5-os-p5 106.787 126.250

o -p5-o 85.510 115.800

o -p5-os 81.819 115.460

os-p5-os 83.949 101.840

p5-os-c3 83.251 119.540

os-c3-c3 85.306 107.970

p5-o-c3 83.251 119.540

os-c3-h1 62.377 109.780

c3-c3-c3 64.888 111.510

c3-c3-h1 46.868 109.560

c3-os-c3 66.293 112.480

c3-c3-oh 84.642 110.190

os-c3-na 109.296 109.030

os-c3-Y1 109.296 109.030

os-c3-h2 62.442 109.580

c3-oh-ho 49.027 107.260

c3-c3-hc 46.816 109.800

oh-c3-h1 62.540 110.260

c3-c3-na 82.668 112.880

c3-c3-h2 46.730 110.220

c3-na-cc 63.695 126.460

c3-Y1-cc 63.695 126.460

na-c3-h2 61.963 107.310

na-cc-h5 61.226 121.550

na-cc-cd 92.653 106.990

Y1-cc-cd 92.653 106.990

na-cc-nc 108.812 121.950

Y1-cc-nc 108.812 121.950

cc-na-cc 70.492 109.900

cd-c -o 86.736 123.930

cd-c -n 87.145 112.700

cd-cc-nc 91.057 111.650

c -cd-cc 67.187 121.350

c -n -cd 66.579 123.270

c -n -hn 48.691 117.550

o -c -n 113.811 123.050

n -cd-nh 110.935 116.940

n -cd-nc 110.304 123.000

cd-n -hn 48.285 119.260

cd-nh-hn 49.267 115.630

cd-nc-cc 73.871 105.490

nh-cd-nc 111.697 120.650

h1-c3-h1 38.802 108.460

hc-c3-hc 38.960 107.580

hn-nh-hn 39.519 115.120

M1-Y1-cd 183.02 128.65 Created by Seminario method using MCPB.py

M1-Y2-hn 65.00 112.03 Created by Seminario method using MCPB.py

M1-Y3-hn 61.05 107.39 Created by Seminario method using MCPB.py

M1-Y4-hn 6.91 75.07 Created by Seminario method using MCPB.py

Y1-M1-Y2 182.93 101.20 Created by Seminario method using MCPB.py

Y1-M1-Y3 183.35 92.13 Created by Seminario method using MCPB.py

Y1-M1-Y4 159.20 174.42 Created by Seminario method using MCPB.py

Y2-M1-Y3 150.16 166.59 Created by Seminario method using MCPB.py

Y2-M1-Y4 141.81 82.68 Created by Seminario method using MCPB.py

Y3-M1-Y4 143.98 83.92 Created by Seminario method using MCPB.py

c3-Y2-M1 159.25 107.57 Created by Seminario method using MCPB.py

na-M1-Y1 182.93 101.20 Created by Seminario method using MCPB.py

na-M1-Y2 183.35 92.13 Created by Seminario method using MCPB.py

na-M1-Y3 159.20 174.42 Created by Seminario method using MCPB.py

na-M1-Y4 150.16 166.59 Created by Seminario method using MCPB.py

c3-Y3-M1 135.52 107.32 Created by Seminario method using MCPB.py

c3-Y4-M1 149.84 109.73 Created by Seminario method using MCPB.py

cc-Y1-M1 152.18 123.14 Created by Seminario method using MCPB.py

cc-na-M1 152.18 123.14 Created by Seminario method using MCPB.py

c3-na-M1 152.18 123.14 Created by Seminario method using MCPB.py

c3-Y1-M1 152.18 123.14 Created by Seminario method using MCPB.py

Y1-cd-c 66.22 123.32 CORR_SOURCE5 27 2.2025

Y1-cd-cc 72.17 111.65 CORR_SOURCE5 1656 1.8430

c3-Y2-hn 47.42 109.29 SOURCE3_SOURCE5 6742 0.6614

c3-Y3-hn 47.42 109.29 SOURCE3_SOURCE5 6742 0.6614

c3-Y4-c3 63.82 112.35 SOURCE3_SOURCE5 10425 1.3688

c3-Y4-hn 47.42 109.29 SOURCE3_SOURCE5 6742 0.6614

c3-c3-Y2 66.02 111.04 SOURCE3_SOURCE5 12086 1.5519

c3-c3-Y3 66.02 111.04 SOURCE3_SOURCE5 12086 1.5519

c3-c3-Y4 66.02 111.04 SOURCE3_SOURCE5 12086 1.5519

c3-c3-Y1 66.02 111.04 SOURCE3_SOURCE5 12086 1.5519

cc-Y1-cd 71.76 105.49 CORR_SOURCE5 1810 1.9032

h1-c3-Y2 49.53 109.88 SOURCE3_SOURCE5 20428 1.2681

h1-c3-Y3 49.53 109.88 SOURCE3_SOURCE5 20428 1.2681

h1-c3-Y4 49.53 109.88 SOURCE3_SOURCE5 20428 1.2681

h5-cc-Y1 50.58 125.52 SOURCE3_SOURCE5 1309 0.7276

hn-Y2-hn 41.40 106.40 SOURCE3_SOURCE5 2019 0.9777

hn-Y3-hn 41.40 106.40 SOURCE3_SOURCE5 2019 0.9777

na-cc-Y1 74.90 112.22 SOURCE3_SOURCE5 2726 1.5103

cc-Y1-cc 71.76 105.49 CORR_SOURCE5 1810 1.9032

Y1-c3-h2 61.963 107.310

DIHE

p5-os-c3-c3 1 0.383 0.000 -3.000

p5-os-p5-o 1 0.800 0.000 2.000

p5-os-p5-os 1 0.800 0.000 2.000

os-p5-os-c3 1 0.000 0.000 -3.000

os-p5-os-c3 1 2.610 0.000 2.000

p5-os-c3-c3 1 3.950 180.000 1.000

p5-os-c3-h1 1 0.217 0.000 3.000

o -p5-os-c3 1 0.800 0.000 -2.000

o -p5-os-c3 1 0.800 0.000 -2.000

o -p5-os-p5 1 0.550 0.000 3.000

o -p5-os-p5 1 0.550 0.000 3.000

os-c3-c3-os 1 0.000 0.000 -3.000

os-c3-c3-os 1 0.000 180.000 -2.000

os-c3-c3-os 1 0.170 180.000 1.000

os-c3-c3-c3 1 0.156 0.000 3.000

os-c3-c3-h1 1 0.000 0.000 -3.000

os-c3-c3-h1 1 0.250 0.000 1.000

c3-c3-os-c3 1 0.240 0.000 -3.000

c3-c3-os-c3 1 0.160 0.000 2.000

c3-c3-c3-oh 1 0.210 0.000 3.000

c3-c3-c3-c3 1 0.130 0.000 -3.000

c3-c3-c3-c3 1 0.290 180.000 -2.000

c3-c3-c3-c3 1 0.110 0.000 1.000

c3-c3-c3-h1 1 0.156 0.000 3.000

c3-os-c3-na 1 0.383 0.000 -3.000

c3-os-c3-na 1 0.650 0.000 2.000

c3-os-c3-h2 1 0.383 0.000 3.000

c3-c3-oh-ho 1 0.000 0.000 3.000

c3-c3-c3-hc 1 0.080 0.000 3.000

os-c3-c3-oh 1 1.010 0.000 -3.000

os-c3-c3-oh 1 0.000 0.000 -2.000

os-c3-c3-oh 1 0.020 180.000 1.000

os-c3-c3-hc 1 0.000 0.000 -3.000

os-c3-c3-hc 1 0.250 0.000 1.000

os-c3-na-cc 1 0.000 0.000 2.000

os-c3-Y1-cc 1 0.000 0.000 2.000

os-c3-Y1-M1 1 0.000 0.000 2.000

c3-c3-c3-na 1 0.156 0.000 3.000

c3-c3-c3-h2 1 0.156 0.000 3.000

oh-c3-c3-h1 1 0.000 0.000 -3.000

oh-c3-c3-h1 1 0.250 0.000 1.000

oh-c3-c3-hc 1 0.180 0.000 -3.000

oh-c3-c3-hc 1 0.510 0.000 1.000

c3-c3-na-cc 1 0.000 0.000 2.000

c3-os-c3-h1 1 0.337 0.000 3.000

#c3-na-cc-nd 1 1.700 180.000 2.000

c3-na-cc-h5 1 1.700 180.000 2.000

c3-na-cc-cd 1 1.700 180.000 2.000

c3-na-cc-nc 1 1.700 180.000 2.000

na-c3-c3-hc 1 0.156 0.000 3.000

na-cc-cd-c 1 4.000 180.000 2.000

na-cc-nc-cd 1 4.750 180.000 2.000

cc-na-c3-h2 1 0.000 0.000 2.000

cc-na-cc-cd 1 1.700 180.000 2.000

cc-na-cc-nc 1 1.700 180.000 2.000

nd-cd-cc-nc 1 4.000 180.000 2.000

cd-c -n -cd 1 2.500 180.000 2.000

cd-c -n -hn 1 2.500 180.000 2.000

cd-cc-nc-cd 1 4.750 180.000 2.000

c -cd-cc-nc 1 4.000 180.000 2.000

c -n -cd-nh 1 1.650 180.000 2.000

c -n -cd-nc 1 1.650 180.000 2.000

o -c -cd-cc 1 2.875 180.000 2.000

o -c -n -cd 1 2.500 180.000 2.000

o -c -n -hn 1 2.500 180.000 -2.000

o -c -n -hn 1 2.000 0.000 1.000

n -c -cd-cc 1 2.875 180.000 2.000

n -cd-nh-hn 1 1.050 180.000 2.000

n -cd-nc-cc 1 4.750 180.000 2.000

nh-cd-n -hn 1 1.650 180.000 2.000

nh-cd-nc-cc 1 4.750 180.000 2.000

nc-cd-n -hn 1 1.650 180.000 2.000

nc-cd-nh-hn 1 1.050 180.000 2.000

cc-na-cc-h5 1 1.700 180.000 2.000

cc-Y1-c3-h2 1 1.700 180.000 2.000

h1-c3-c3-h1 1 0.156 0.000 3.000

h1-c3-oh-ho 1 0.113 0.000 3.000

h1-c3-c3-hc 1 0.156 0.000 3.000

hc-c3-c3-h2 1 0.156 0.000 3.000

X -Y1-cd-X 2 9.5 180.0 2.0 statistiv value from parm94

X -c3-Y2-X 6 1.8 0.0 3.0 Junmei et al, 1999

X -c3-Y3-X 6 1.8 0.0 3.0 Junmei et al, 1999

X -c3-Y4-X 6 1.8 0.0 3.0 Junmei et al, 1999

X -cc-Y1-X 2 9.5 180.0 2.0 statistic value from parm94

M1-Y1-cd-c 3 0.00 0.00 3.0 Treat as zero by MCPB.py

M1-Y1-cd-cc 3 0.00 0.00 3.0 Treat as zero by MCPB.py

Y1-M1-Y2-c3 3 0.00 0.00 3.0 Treat as zero by MCPB.py

Y1-M1-Y2-hn 3 0.00 0.00 3.0 Treat as zero by MCPB.py

Y1-M1-Y3-c3 3 0.00 0.00 3.0 Treat as zero by MCPB.py

Y1-M1-Y3-hn 3 0.00 0.00 3.0 Treat as zero by MCPB.py

Y1-M1-Y4-c3 3 0.00 0.00 3.0 Treat as zero by MCPB.py

Y1-M1-Y4-hn 3 0.00 0.00 3.0 Treat as zero by MCPB.py

Y2-M1-Y1-cd 3 0.00 0.00 3.0 Treat as zero by MCPB.py

Y2-M1-Y3-hn 3 0.00 0.00 3.0 Treat as zero by MCPB.py

Y2-M1-Y4-c3 3 0.00 0.00 3.0 Treat as zero by MCPB.py

Y2-M1-Y4-hn 3 0.00 0.00 3.0 Treat as zero by MCPB.py

Y3-M1-Y1-cd 3 0.00 0.00 3.0 Treat as zero by MCPB.py

Y3-M1-Y2-hn 3 0.00 0.00 3.0 Treat as zero by MCPB.py

Y3-M1-Y4-c3 3 0.00 0.00 3.0 Treat as zero by MCPB.py

Y3-M1-Y4-hn 3 0.00 0.00 3.0 Treat as zero by MCPB.py

Y4-M1-Y1-cd 3 0.00 0.00 3.0 Treat as zero by MCPB.py

Y4-M1-Y2-hn 3 0.00 0.00 3.0 Treat as zero by MCPB.py

Y4-M1-Y3-hn 3 0.00 0.00 3.0 Treat as zero by MCPB.py

c3-Y2-M1-Y3 3 0.00 0.00 3.0 Treat as zero by MCPB.py

c3-na-M1-Y3 3 0.00 0.00 3.0 Treat as zero by MCPB.py

c3-na-M1-Y4 3 0.00 0.00 3.0 Treat as zero by MCPB.py

c3-na-M1-Y2 3 0.00 0.00 3.0 Treat as zero by MCPB.py

c3-Y1-M1-Y3 3 0.00 0.00 3.0 Treat as zero by MCPB.py

c3-Y2-M1-Y4 3 0.00 0.00 3.0 Treat as zero by MCPB.py

c3-Y3-M1-Y2 3 0.00 0.00 3.0 Treat as zero by MCPB.py

c3-Y3-M1-Y4 3 0.00 0.00 3.0 Treat as zero by MCPB.py

c3-c3-Y2-M1 3 0.00 0.00 3.0 Treat as zero by MCPB.py

c3-c3-Y3-M1 3 0.00 0.00 3.0 Treat as zero by MCPB.py

c3-c3-Y4-M1 3 0.00 0.00 3.0 Treat as zero by MCPB.py

c3-c3-Y1-M1 3 0.00 0.00 3.0 Treat as zero by MCPB.py

c3-c3-Y4-c3 1 0.3 0.0 -3.0 Junmei et al, 1999

c3-c3-Y4-c3 1 0.48 180.0 2.0 Junmei et al, 1999

c3-c3-Y1-cc 1 0.48 180.0 2.0 Junmei et al, 1999

cc-Y1-M1-Y2 3 0.00 0.00 3.0 Treat as zero by MCPB.py

cc-Y1-M1-Y3 3 0.00 0.00 3.0 Treat as zero by MCPB.py

cc-Y1-M1-Y4 3 0.00 0.00 3.0 Treat as zero by MCPB.py

h1-c3-Y2-M1 3 0.00 0.00 3.0 Treat as zero by MCPB.py

h1-c3-Y3-M1 3 0.00 0.00 3.0 Treat as zero by MCPB.py

h1-c3-Y4-M1 3 0.00 0.00 3.0 Treat as zero by MCPB.py

h2-c3-Y1-M1 3 0.00 0.00 3.0 Treat as zero by MCPB.py

h5-cc-Y1-M1 3 0.00 0.00 3.0 Treat as zero by MCPB.py

na-cc-Y1-M1 3 0.00 0.00 3.0 Treat as zero by MCPB.py

na-cc-cd-Y1 3 0.00 0.00 3.0

Y1-cc-na-cc 3 0.00 0.00 3.0

Y1-cd-c-o 3 0.00 0.00 3.0

Y1-cd-c-n 3 0.00 0.00 3.0

Y1-cd-cc-nc 3 0.00 0.00 3.0

Y2-c3-c3-Y4 3 0.00 0.00 3.0

Y2-c3-c3-h1 3 0.00 0.00 3.0

Y2-c3-c3-h1 3 0.00 0.00 3.0

Y3-c3-c3-Y4 3 0.00 0.00 3.0

Y3-c3-c3-h1 3 0.00 0.00 3.0

Y3-c3-c3-h1 3 0.00 0.00 3.0

Y4-c3-c3-h1 3 0.00 0.00 3.0

Y4-c3-c3-h1 3 0.00 0.00 3.0

Y4-c3-c3-h1 3 0.00 0.00 3.0

Y4-c3-c3-h1 3 0.00 0.00 3.0

c3-na-cc-Y1 3 0.00 0.00 3.0

IMPROPER

c3-cc-na-cc 1.1 180.0 2.0 Using default value

cd-n -c -o 10.5 180.0 2.0 General improper torsional angle (2 general atom types)

c -cd-n -hn 1.1 180.0 2.0 General improper torsional angle (2 general atom types)

n -nc-cd-nh 1.1 180.0 2.0 Using default value

cd-hn-nh-hn 1.1 180.0 2.0 Using default value

cd-na-cc-nc 1.1 180.0 2.0 Using default value

Y1-h5-cc-na 1.1 180.0 2.0 Using default value

Y1-c -cd-cc 1.1 180.0 2.0 Using default value

NONBON

p5 2.0732 0.2295

o 1.7107 0.1463

os 1.7713 0.0726

c3 1.9069 0.1078

oh 1.8200 0.0930

na 1.7992 0.2042

cc 1.8606 0.0988

cd 1.8606 0.0988

c 1.8606 0.0988

n 1.7852 0.1636

nh 1.7903 0.2150

nc 1.8993 0.0941

h1 1.3593 0.0208

hc 1.4593 0.0208

h2 1.2593 0.0208

h5 1.3735 0.0161

hn 0.6210 0.0100

ho 0.3019 0.0047

M1 1.2660 0.0030764200 CM set for Pt2+ ion in TIP3P water from Li et al. JCTC, 2013, 9, 2733

Y1 1.8240 0.1700 OPLS

Y2 1.8240 0.1700 OPLS

Y3 1.8240 0.1700 OPLS

Y4 1.8240 0.1700 OPLS

Parameters complex 2

File off:

!!index array str

"MOL"

!entry.MOL.unit.atoms table str name str type int typex int resx int flags int seq int elmnt dbl chg

"P1" "p5" 0 1 131072 1 15 1.125285

"O1" "o" 0 1 131072 2 8 -0.754291

"O2" "oh" 0 1 131072 3 8 -0.604601

"O3" "o" 0 1 131072 4 8 -0.754291

"O4" "os" 0 1 131072 5 8 -0.418199

"P2" "p5" 0 1 131072 6 15 1.098280

"O5" "o" 0 1 131072 7 8 -0.716928

"O6" "o" 0 1 131072 8 8 -0.716928

"O7" "os" 0 1 131072 9 8 -0.469240

"P3" "p5" 0 1 131072 10 15 1.333113

"O8" "oh" 0 1 131072 11 8 -0.813652

"O9" "o" 0 1 131072 12 8 -0.743769

"O10" "os" 0 1 131072 13 8 -0.393679

"C1" "c3" 0 1 131072 14 6 -0.177885

"C2" "c3" 0 1 131072 15 6 0.367282

"O11" "os" 0 1 131072 16 8 -0.527228

"C3" "c3" 0 1 131072 17 6 0.531252

"O12" "oh" 0 1 131072 18 8 -0.773339

"C4" "c3" 0 1 131072 19 6 -0.304723

"C5" "c3" 0 1 131072 20 6 0.244592

"N1" "na" 0 1 131072 21 7 -0.241174

"C6" "c2" 0 1 131072 22 6 0.328615

"N2" "Y1" 0 1 131072 23 7 -0.363914

"C7" "c2" 0 1 131072 24 6 -0.322051

"C8" "c2" 0 1 131072 25 6 0.877511

"O13" "o" 0 1 131072 26 8 -0.665214

"N3" "ns" 0 1 131072 27 7 -0.828823

"C9" "c2" 0 1 131072 28 6 0.997780

"N4" "nv" 0 1 131072 29 7 -0.993401

"N5" "n2" 0 1 131072 30 7 -0.797741

"C10" "c2" 0 1 131072 31 6 0.581560

"N6" "Y2" 0 1 131072 32 7 -0.944276

"N7" "Y3" 0 1 131072 33 7 -1.215885

"H1" "h1" 0 1 131072 34 1 0.107442

"H2" "h1" 0 1 131072 35 1 0.107442

"H3" "h1" 0 1 131072 36 1 -0.013563

"H4" "h1" 0 1 131072 37 1 -0.057286

"H5" "hc" 0 1 131072 38 1 0.095972

"H6" "hc" 0 1 131072 39 1 0.095972

"H7" "h2" 0 1 131072 40 1 0.092949

"H8" "h5" 0 1 131072 41 1 0.192431

"H9" "hn" 0 1 131072 42 1 0.417336

"H10" "ho" 0 1 131072 43 1 0.342319

"H11" "hn" 0 1 131072 44 1 0.399656

"H12" "hn" 0 1 131072 45 1 0.399656

"H13" "hn" 0 1 131072 46 1 0.392561

"H14" "hn" 0 1 131072 47 1 0.392561

"H15" "ho" 0 1 131072 48 1 0.436381

"PT" "M1" 0 1 131072 49 -1 0.531600

"H16" "hn" 0 1 131072 50 1 0.392561

"H17" "hn" 0 1 131072 51 1 0.363593

"H18" "hn" 0 1 131072 52 1 0.363593

"O14" "Y4" 0 1 131072 53 8 -1.039336

"H19" "ho" 0 1 131072 54 1 0.472268

"H20" "ho" 0 1 131072 55 1 0.569852

!entry.MOL.unit.atomspertinfo table str pname str ptype int ptypex int pelmnt dbl pchg

"P1" "p5" 0 -1 0.0

"O1" "o" 0 -1 0.0

"O2" "oh" 0 -1 0.0

"O3" "o" 0 -1 0.0

"O4" "os" 0 -1 0.0

"P2" "p5" 0 -1 0.0

"O5" "o" 0 -1 0.0

"O6" "o" 0 -1 0.0

"O7" "os" 0 -1 0.0

"P3" "p5" 0 -1 0.0

"O8" "oh" 0 -1 0.0

"O9" "o" 0 -1 0.0

"O10" "os" 0 -1 0.0

"C1" "c3" 0 -1 0.0

"C2" "c3" 0 -1 0.0

"O11" "os" 0 -1 0.0

"C3" "c3" 0 -1 0.0

"O12" "oh" 0 -1 0.0

"C4" "c3" 0 -1 0.0

"C5" "c3" 0 -1 0.0

"N1" "na" 0 -1 0.0

"C6" "c2" 0 -1 0.0

"N2" "Y2" 0 -1 0.0

"C7" "c2" 0 -1 0.0

"C8" "c2" 0 -1 0.0

"O13" "o" 0 -1 0.0

"N3" "ns" 0 -1 0.0

"C9" "c2" 0 -1 0.0

"N4" "nv" 0 -1 0.0

"N5" "n2" 0 -1 0.0

"C10" "c2" 0 -1 0.0

"N6" "Y2" 0 -1 0.0

"N7" "Y3" 0 -1 0.0

"H1" "h1" 0 -1 0.0

"H2" "h1" 0 -1 0.0

"H3" "h1" 0 -1 0.0

"H4" "h1" 0 -1 0.0

"H5" "hc" 0 -1 0.0

"H6" "hc" 0 -1 0.0

"H7" "h2" 0 -1 0.0

"H8" "h5" 0 -1 0.0

"H9" "hn" 0 -1 0.0

"H10" "ho" 0 -1 0.0

"H11" "hn" 0 -1 0.0

"H12" "hn" 0 -1 0.0

"H13" "hn" 0 -1 0.0

"H14" "hn" 0 -1 0.0

"H15" "ho" 0 -1 0.0

"PT" "M1" 0 -1 0.0

"H16" "hn" 0 -1 0.0

"H17" "hn" 0 -1 0.0

"H18" "hn" 0 -1 0.0

"O14" "Y4" 0 -1 0.0

"H19" "ho" 0 -1 0.0

"H20" "ho" 0 -1 0.0

!entry.MOL.unit.boundbox array dbl

-1.000000

0.0

0.0

0.0

0.0

!entry.MOL.unit.childsequence single int

2

!entry.MOL.unit.connect array int

0

0

!entry.MOL.unit.connectivity table int atom1x int atom2x int flags

1 2 1

1 3 1

1 4 1

1 5 1

5 6 1

6 7 1

6 8 1

6 9 1

9 10 1

10 11 1

10 12 1

10 13 1

13 14 1

14 15 1

14 34 1

14 35 1

15 16 1

15 17 1

15 36 1

16 20 1

17 18 1

17 19 1

17 37 1

18 48 1

19 20 1

19 38 1

19 39 1
20 21 1
20 40 1
21 22 1
21 31 1
22 23 1
22 41 1
23 24 1
23 49 1
24 25 1
24 31 1
25 26 1
25 27 1
27 28 1
27 42 1
28 29 1
28 30 1
29 44 1
29 45 1
30 31 1
32 46 1
32 47 1
32 50 1
32 49 1
33 51 1
33 52 1
33 43 1
33 49 1
49 53 1
53 54 1
53 55 1

!entry.MOL.unit.hierarchy table str abovetype int abovex str belowtype int belowx

"U" 0 "R" 1
"R" 1 "A" 1
"R" 1 "A" 2
"R" 1 "A" 3
"R" 1 "A" 4
"R" 1 "A" 5
"R" 1 "A" 6
"R" 1 "A" 7
"R" 1 "A" 8
"R" 1 "A" 9
"R" 1 "A" 10
"R" 1 "A" 11
"R" 1 "A" 12
"R" 1 "A" 13
"R" 1 "A" 14
"R" 1 "A" 15
"R" 1 "A" 16
"R" 1 "A" 17
"R" 1 "A" 18
"R" 1 "A" 19
"R" 1 "A" 20
"R" 1 "A" 21
"R" 1 "A" 22

"R" 1 "A" 23
"R" 1 "A" 24
"R" 1 "A" 25
"R" 1 "A" 26
"R" 1 "A" 27
"R" 1 "A" 28
"R" 1 "A" 29
"R" 1 "A" 30
"R" 1 "A" 31
"R" 1 "A" 32
"R" 1 "A" 33
"R" 1 "A" 34
"R" 1 "A" 35
"R" 1 "A" 36
"R" 1 "A" 37
"R" 1 "A" 38
"R" 1 "A" 39
"R" 1 "A" 40
"R" 1 "A" 41
"R" 1 "A" 42
"R" 1 "A" 43
"R" 1 "A" 44
"R" 1 "A" 45
"R" 1 "A" 46
"R" 1 "A" 47
"R" 1 "A" 48
"R" 1 "A" 49
"R" 1 "A" 50
"R" 1 "A" 51
"R" 1 "A" 52
"R" 1 "A" 53
"R" 1 "A" 54
"R" 1 "A" 55
!entry.MOL.unit.name single str
"MOL"
!entry.MOL.unit.positions table dbl x dbl y dbl z
0.636000 -0.359000 2.908000
-0.713000 -0.039000 2.264000
1.141000 -1.828000 2.340000
0.783000 -0.328000 4.394000
1.714000 0.674000 2.140000
3.256000 0.511000 1.609000
3.611000 1.842000 0.971000
4.165000 -0.191000 2.552000
2.913000 -0.581000 0.313000
3.154000 -0.406000 -1.269000
2.505000 -1.690000 -1.894000
4.540000 -0.100000 -1.705000
2.090000 0.784000 -1.710000
2.580000 2.085000 -2.029000
1.574000 3.147000 -1.630000
0.317000 2.995000 -2.364000
1.182000 3.197000 -0.140000
2.091000 3.923000 0.634000
-0.199000 3.857000 -0.225000
-0.766000 3.239000 -1.506000

-1.459000 1.949000 -1.224000

-0.957000 0.693000 -1.440000

-1.747000 -0.246000 -0.949000

-2.819000 0.420000 -0.384000

-3.998000 -0.044000 0.287000

-4.421000 -1.172000 0.527000

-4.785000 1.072000 0.715000

-4.486000 2.395000 0.534000

-5.409000 3.314000 1.038000

-3.432000 2.813000 -0.110000

-2.638000 1.796000 -0.549000

-1.753000 -2.311000 1.216000

-0.033000 -4.003000 -0.355000

2.778000 2.141000 -3.109000

3.508000 2.288000 -1.487000

2.003000 4.126000 -1.897000

1.073000 2.175000 0.241000

-0.076000 4.938000 -0.367000

-0.827000 3.682000 0.650000

-1.506000 3.871000 -2.012000

-0.004000 0.519000 -1.914000

-5.644000 0.802000 1.176000

1.464000 -1.735000 1.427000

-5.010000 4.245000 0.991000

-5.711000 3.103000 1.983000

-1.442000 -3.150000 1.699000

-1.392000 -1.458000 1.735000

2.743000 3.224000 0.931000

-0.909000 -2.200000 -0.671000

-2.772000 -2.296000 1.167000

0.410000 -3.927000 0.567000

0.783000 -4.012000 -0.974000

0.112000 -1.963000 -2.462000

0.080000 -2.824000 -2.899000

1.456000 -1.750000 -2.108000

!entry.MOL.unit.residueconnect table int c1x int c2x int c3x int c4x int c5x int c6x

0 0 0 0 0 0

!entry.MOL.unit.residues table str name int seq int childseq int startatomx str restype int imagingx

"MOL" 1 56 1 "?" 0

!entry.MOL.unit.residuesPdbSequenceNumber array int

0

!entry.MOL.unit.solventcap array dbl

-1.000000

0.0

0.0

0.0

0.0

!entry.MOL.unit.velocities table dbl x dbl y dbl z

0.0 0.0 0.0

0.0 0.0 0.0

0.0 0.0 0.0

0.0 0.0 0.0

0.0 0.0 0.0

0.0 0.0 0.0

0.0 0.0 0.0

0.0 0.0 0.0
0.0 0.0 0.0
0.0 0.0 0.0
0.0 0.0 0.0
0.0 0.0 0.0
0.0 0.0 0.0
0.0 0.0 0.0
0.0 0.0 0.0
0.0 0.0 0.0
0.0 0.0 0.0
0.0 0.0 0.0
0.0 0.0 0.0
0.0 0.0 0.0
0.0 0.0 0.0
0.0 0.0 0.0
0.0 0.0 0.0
0.0 0.0 0.0
0.0 0.0 0.0
0.0 0.0 0.0
0.0 0.0 0.0
0.0 0.0 0.0
0.0 0.0 0.0
0.0 0.0 0.0
0.0 0.0 0.0
0.0 0.0 0.0
0.0 0.0 0.0
0.0 0.0 0.0
0.0 0.0 0.0

0.0 0.0 0.0
0.0 0.0 0.0
0.0 0.0 0.0
0.0 0.0 0.0
0.0 0.0 0.0
0.0 0.0 0.0
0.0 0.0 0.0
0.0 0.0 0.0
0.0 0.0 0.0
0.0 0.0 0.0
0.0 0.0 0.0
0.0 0.0 0.0
0.0 0.0 0.0
0.0 0.0 0.0
0.0 0.0 0.0
0.0 0.0 0.0
0.0 0.0 0.0
0.0 0.0 0.0
0.0 0.0 0.0
0.0 0.0 0.0

File frcmod:

remark gY4es here
MASS
p5 30.970 1.538
oh 16.000 0.465
os 16.000 0.465
c3 12.010 0.878
na 14.010 0.530

c2 12.010 0.360

n2 14.010 0.530

ns 14.010 0.530

nv 14.010 0.530

n9 14.010 0.530

h1 1.008 0.135

hc 1.008 0.135

h2 1.008 0.135

h5 1.008 0.135

hn 1.008 0.161

ho 1.008 0.135

M1 195.08 Pt iY4n

Y1 14.010 0.530

Y2 14.010 0.530

Y3 14.010 0.530

Y4 16.000 0.434

BOND

p5-Y4 529.55 1.487

p5-oh 346.03 1.615

p5-os 346.25 1.615

oh-ho 563.51 0.973

os-c3 284.76 1.432

c3-c3 232.52 1.538

c3-h1 375.92 1.097

c3-oh 293.40 1.423

c3-hc 375.92 1.097

c3-na 262.85 1.463

c3-h2 377.33 1.096

na-c2 327.66 1.401

c2-n2 518.67 1.282

c2-h5 386.13 1.091

c2-c2 481.83 1.334

c2-Y4 635.23 1.225

c2-ns 330.19 1.399 same as c2-n

ns-hn 527.31 1.013 same as hn-n

c2-nv 345.39 1.387 same as c2-nh

nv-hn 529.46 1.012 same as hn-nh

n9-hn 511.28 1.019 same as hn-n3

n2-hn 501.09 1.023

Y4 -ho 540.28 0.981

Y1-M1 80.3 2.1471 Created by SeminariY4 method using MCPB.py

Y2-M1 80.3 2.1471 Created by SeminariY4 method using MCPB.py

Y3-M1 80.0 2.1471 Created by SeminariY4 method using MCPB.py

Y4-M1 78.6 2.3471 Created by SeminariY4 method using MCPB.py

Y4 -M1 78.6 2.6178 Created by Seminario method using MCPB.py

Y2-hn 511.28 1.019 same as hn-n3

Y3-hn 501.09 1.023

Y3-ho 501.09 1.023

Y4-ho 540.28 0.981

c2-Y1 518.67 1.282

oh-M1 81.6 2.6178

ANGLE

p5-oh-ho 58.997 110.080

p5-oh-M1 58.997 110.080

p5-Y4 -M1 58.997 110.080

p5-os-p5 106.787 126.250

Y4 -p5-oh 81.901 115.210

Y4 -p5-o 85.510 115.800

Y4 -p5-os 81.819 115.460

oh-p5-os 83.902 101.940

os-p5-os 83.949 101.840

p5-os-c3 83.251 119.540

os-c3-c3 85.306 107.970

os-c3-h1 62.377 109.780

c3-c3-c3 64.888 111.510

c3-c3-h1 46.868 109.560

c3-os-c3 66.293 112.480

c3-c3-oh 84.642 110.190

os-c3-na 109.296 109.030

o -c2-ns 109.296 109.030

os-c3-h2 62.442 109.580

c3-oh-ho 49.027 107.260

c3-c3-hc 46.816 109.800

oh-c3-h1 62.540 110.260

c3-c3-na 82.668 112.880

c3-c3-h2 46.730 110.220

c3-na-c2 65.723 117.200

na-c3-h2 61.963 107.310

na-c2-n2 110.312 123.620

na-c2-h5 59.208 126.390

na-c2-c2 87.232 121.940

c2-na-c2 69.273 110.370

c2-n2-c2 73.202 118.180

n2-c2-h5 64.543 121.700

n2-c2-c2 89.778 126.010

c2-c2-Y4 89.793 130.890

c2-c2-ns 86.693 123.670 same as c2-c2-n

c2-c2-c2 71.539 121.810

c2-ns-c2 67.455 116.750 same as c2-n -c2

c2-ns-hn 47.988 117.900 same as c2-n -hn

Y4 -c2-ns 75.107 117.460 Calculated with empirical approach

ns-c2-nv 113.384 109.350 same as n -c2-nh

ns-c2-n2 110.772 122.820 same as n -c2-n2

c2-nv-hn 48.954 115.090 same as c2-nh-hn

nv-c2-n2 110.708 124.270 same as n2-c2-nh

h1-c3-h1 38.802 108.460

hc-c3-hc 38.960 107.580

hn-nv-hn 39.519 115.120 same as hn-nh-hn

hn-n9-hn 40.828 106.400 same as hn-n3-hn

hn-n2-hn 38.294 120.000

M1-Y2-hn 67.55 104.19 Created by SeminariY4 method using MCPB.py

M1-Y3-hn 67.39 104.19 Created by SeminariY4 method using MCPB.py

M1-Y3-ho 67.39 104.19

M1-Y4-ho 50.01 91.15 Created by Seminario method using MCPB.py

Y1-M1-Y2 182.00 90.94 Created by SeminariY4 method using MCPB.py

Y1-M1-Y3 182.48 180.46 Created by SeminariY4 method using MCPB.py

Y1-M1-Y4 182.50 89.61 Created by SeminariY4 method using MCPB.py

Y2-M1-Y3 182.00 87.62 Created by SeminariY4 method using MCPB.py

Y2-M1-Y4 182.43 183.80 Created by SeminariY4 method using MCPB.py

Y3-M1-Y4 182.71 96.43 Created by SeminariY4 method using MCPB.py

oh-M1-Y2 180.43 83.80 Created by Seminario method using MCPB.py

oh-M1-Y3 182.43 83.80 Created by Seminario method using MCPB.py

oh-M1-Y1 182.43 93.80 Created by Seminario method using MCPB.py

oh-M1-Y4 182.71 86.43 Created by Seminario method using MCPB.py

ho -oh-M1 72.71 86.43 Created by Seminario method using MCPB.py

Y4 -M1-oh 72.71 76.43 Created by Seminario method using MCPB.py

c2-Y1-M1 11.11 120.68 Created by SeminariY4 method using MCPB.py

Y1-c2-c2 89.778 126.010

c2-Y1-c2 73.202 118.180

h5-c2-Y1 52.26 121.70 SOURCE4_SOURCE5 71 2.1538

hn-Y2-hn 40.828 106.400 same as hn-n3-hn

hn-Y3-hn 38.294 120.000

ho-Y3-hn 38.294 120.000

na-c2-Y1 110.312 123.620

ho-Y4-ho 38.294 120.000

DIHE

p5-os-p5-o 1 0.800 0.000 2.000

p5-os-p5-os 1 0.800 0.000 2.000

Y4 -p5-oh-ho 1 0.367 0.000 3.000

oh-p5-os-p5 1 0.800 0.000 2.000

os-p5-oh-ho 1 0.533 0.000 3.000

os-p5-os-c3 1 0.000 0.000 -3.000

os-p5-os-c3 1 2.610 0.000 2.000

p5-os-c3-c3 1 0.383 0.000 -3.000

p5-os-c3-c3 1 3.950 180.000 1.000

p5-os-c3-h1 1 0.217 0.000 3.000

oh-p5-os-c3 1 0.250 0.000 -3.000

oh-p5-os-c3 1 1.200 0.000 2.000

Y4 -p5-os-c3 1 0.800 0.000 -2.000

Y4 -p5-os-c3 1 0.550 0.000 3.000

os-c3-c3-os 1 0.000 0.000 -3.000

os-c3-c3-os 1 0.000 180.000 -2.000

os-c3-c3-os 1 0.170 180.000 1.000

os-c3-c3-c3 1 0.156 0.000 3.000

os-c3-c3-h1 1 0.000 0.000 -3.000

os-c3-c3-h1 1 0.250 0.000 1.000

c3-c3-os-c3 1 0.240 0.000 -3.000

c3-c3-os-c3 1 0.160 0.000 2.000

c3-c3-c3-oh 1 0.210 0.000 3.000

c3-c3-c3-c3 1 0.130 0.000 -3.000

c3-c3-c3-c3 1 0.290 180.000 -2.000

c3-c3-c3-c3 1 0.110 0.000 1.000

c3-c3-c3-h1 1 0.156 0.000 3.000

c3-os-c3-na 1 0.383 0.000 -3.000

c3-os-c3-na 1 0.650 0.000 2.000

c3-os-c3-h2 1 0.383 0.000 3.000

c3-c3-oh-ho 1 0.000 0.000 3.000

c3-c3-c3-hc 1 0.080 0.000 3.000

os-c3-c3-oh 1 1.010 0.000 -3.000

os-c3-c3-oh 1 0.000 0.000 -2.000

os-c3-c3-oh 1 0.020 180.000 1.000

os-c3-c3-hc 1 0.000 0.000 -3.000

os-c3-c3-hc 1 0.250 0.000 1.000

os-c3-na-c2 1 0.000 0.000 -2.000

os-c3-na-c2 1 2.500 0.000 1.000

c3-c3-c3-na 1 0.156 0.000 3.000

c3-c3-c3-h2 1 0.156 0.000 3.000

oh-c3-c3-h1 1 0.000 0.000 -3.000

oh-c3-c3-h1 1 0.250 0.000 1.000

oh-c3-c3-hc 1 0.180 0.000 -3.000

oh-c3-c3-h 1 0.510 0.000 1.000

c3-c3-na-c2 1 0.000 0.000 2.000

c3-os-c3-h1 1 0.337 0.000 3.000

c3-na-c2-n2 1 0.625 180.000 2.000

c3-na-c2-h5 1 0.625 180.000 2.000

c3-na-c2-c2 1 0.625 180.000 2.000

na-c3-c3-hc 1 0.156 0.000 3.000

na-c2-n2-c2 1 4.150 180.000 2.000

na-c2-c2-n2 1 6.650 180.000 2.000

na-c2-c2-c2 1 6.650 180.000 2.000

c2-na-c3-h2 1 0.000 0.000 2.000

c2-na-c2-c2 1 0.625 180.000 2.000

c2-na-c2-n2 1 0.625 180.000 2.000

c2-n2-c2-c2 1 4.150 180.000 2.000

n2-c2-c2-Y4 1 6.650 180.000 2.000

n2-c2-c2-ns 1 6.650 180.000 2.000

n2-c2-c2-n2 1 6.650 180.000 2.000

c2-n2-c2-h5 1 4.150 180.000 2.000

c2-c2-ns-c2 1 0.650 180.000 2.000 same as X -c2-n -X

c2-c2-ns-hn 1 0.650 180.000 2.000 same as X -c2-n -X

c2-c2-c2-n2 1 6.650 180.000 2.000

c2-ns-c2-nv 1 0.650 180.000 2.000 same as X -c2-n -X

c2-ns-c2-n2 1 0.650 180.000 2.000 same as X -c2-n -X

Y4 -c2-c2-c2 1 6.650 180.000 2.000

Y4 -c2-ns-c2 1 0.650 180.000 2.000 same as X -c2-n -X

Y4 -c2-ns-hn 1 0.650 180.000 2.000 same as X -c2-n -X

ns-c2-c2-c2 1 6.650 180.000 2.000

ns-c2-nv-hn 1 0.675 180.000 2.000 same as X -c2-nh-X

ns-c2-n2-c2 1 4.150 180.000 2.000

nv-c2-ns-hn 1 0.650 180.000 2.000 same as X -c2-n -X

nv-c2-n2-c2 1 4.150 180.000 2.000

n2-c2-ns-hn 1 0.650 180.000 2.000 same as X -c2-n -X

n2-c2-nv-hn 1 0.675 180.000 2.000 same as X -c2-nh-X

c2-na-c2-h5 1 0.625 180.000 2.000

h1-c3-c3-h1 1 0.156 0.000 3.000

h1-c3-oh-ho 1 0.113 0.000 3.000

h1-c3-c3-hc 1 0.156 0.000 3.000

hc-c3-c3-h2 1 0.156 0.000 3.000

M1-Y1-c2-c2 3 0.00 0.00 3.0 Treat as zerY4 by MCPB.py

Y1-M1-Y2-hn 3 0.00 0.00 3.0 Treat as zerY4 by MCPB.py

oh-M1-Y2-hn 3 0.00 0.00 3.0 Treat as zero by MCPB.py

oh-M1-Y3-hn 3 0.00 0.00 3.0 Treat as zero by MCPB.py

oh-M1-Y1-c2 3 0.00 0.00 3.0 Treat as zero by MCPB.py

oh-M1-Y4-ho 3 0.00 0.00 3.0 Treat as zero by MCPB.py

Y1-M1-Y3-hn 3 0.00 0.00 3.0 Treat as zerY4 by MCPB.py

Y1-M1-Y4-ho 3 0.00 0.00 3.0 Treat as zero by MCPB.py

Y1-c2-c2-n2 1 6.65 180.0 2.0

Y1-c2-c2-ns 1 6.65 180.0 2.0

Y1-c2-c2-Y4 1 6.65 180.0 2.0

Y2-M1-Y1-c2 3 0.00 0.00 3.0 Treat as zerY4 by MCPB.py

Y2-M1-Y3-hn 3 0.00 0.00 3.0 Treat as zerY4 by MCPB.py

Y2-M1-Y4-ho 3 0.00 0.00 3.0 Treat as zero by MCPB.py

Y3-M1-Y1-c2 3 0.00 0.00 3.0 Treat as zerY4 by MCPB.py

Y3-M1-Y2-hn 3 0.00 0.00 3.0 Treat as zerY4 by MCPB.py

Y3-M1-Y4-ho 3 0.00 0.00 3.0 Treat as zero by MCPB.py

Y4-M1-Y1-c2 3 0.00 0.00 3.0 Treat as zerY4 by MCPB.py

Y4-M1-Y2-hn 3 0.00 0.00 3.0 Treat as zerY4 by MCPB.py

Y4-M1-Y3-hn 3 0.00 0.00 3.0 Treat as zerY4 by MCPB.py

c2-Y1-M1-Y2 3 0.00 0.00 3.0 Treat as zerY4 by MCPB.py

c2-Y1-M1-Y3 3 0.00 0.00 3.0 Treat as zerY4 by MCPB.py

c2-Y1-M1-Y4 3 0.00 0.00 3.0 Treat as zerY4 by MCPB.py

c2-Y1-c2-c2 1 4.15 180.0 2.0

c2-na-c2-Y1 1 0.625 180.0 2.0

c3-na-c2-Y1 1 0.625 180.0 2.0

h5-c2-Y1-M1 3 0.00 0.00 3.0 Treat as zerY4 by MCPB.py

h5-c2-Y1-c2 1 4.15 180.0 2.0

na-c2-Y1-M1 3 0.00 0.00 3.0 Treat as zerY4 by MCPB.py

na-c2-Y1-c2 1 4.15 180.0 2.0

na-c2-c2-Y1 1 6.65 180.0 2.0

ho-Y3-M1-Y4 3 0.00 0.00 3.0

Y2-M1-Y3-ho 3 0.00 0.00 3.0

Y1-M1-Y3-ho 3 0.00 0.00 3.0

o -c2-ns-c2 3 0.00 0.00 3.0

o -c2-ns-hn 3 0.00 0.00 3.0

IMPROPER

c2-c2-na-c3 1.1 180.0 2.0

h5-n2-c2-na 1.1 180.0 2.0 Using default value

c2-c2-c2-n2 1.1 180.0 2.0 Using default value

c2-ns-c2-Y4 1.1 180.0 2.0 Using default value

c2-c2-ns-hn 1.1 180.0 2.0 Using default value

n2-ns-c2-nv 1.1 180.0 2.0 Using default value

c2-hn-nv-hn 1.1 180.0 2.0 Using default value

c2-n2-c2-na 1.1 180.0 2.0 Using default value

Y1-c2-c2-c2 1.1 180.0 2.0 Using default value

Y1-h5-c2-na 1.1 180.0 2.0 Using default value

NONBON

p5 2.0732 0.2295

Y4 1.7107 0.1463

oh 1.8200 0.0930

os 1.7713 0.0726

c3 1.9069 0.1078

na 1.7992 0.2042

c2 1.8606 0.0988

n2 1.8993 0.0941

ns 1.8352 0.1174

nv 1.8903 0.1120

n9 2.2700 0.0095

h1 1.3593 0.0208

hc 1.4593 0.0208

h2 1.2593 0.0208

h5 1.3735 0.0161

hn 0.6210 0.0100

ho 0.3019 0.0047

M1 1.2660 0.0030764200 CM set fY4r Pt2+ ion in TIP3P water from Li et al. JCTC, 2013, 9, 2733

Y1 1.8993 0.0941

Y2 2.2700 0.0095

Y3 1.8993 0.0941

Parameters complex 3

File off:

!!index array str

"MOL"

!entry.MOL.unit.atoms table str name str type int typex int resx int flags int seq int elmnt dbl chg

"P1" "p5" 0 1 131072 1 15 1.342234

"O1" "o" 0 1 131072 2 8 -0.739517

"O2" "oh" 0 1 131072 3 8 -0.845220

"O3" "o" 0 1 131072 4 8 -0.845220

"O4" "os" 0 1 131072 5 8 -0.592939

"P2" "p5" 0 1 131072 6 15 1.252046

"O5" "o" 0 1 131072 7 8 -0.795894

"O6" "o" 0 1 131072 8 8 -0.795894

"O7" "os" 0 1 131072 9 8 -0.476918

"P3" "p5" 0 1 131072 10 15 1.117531

"O8" "oh" 0 1 131072 11 8 -0.750455

"O9" "o" 0 1 131072 12 8 -0.750455

"O10" "os" 0 1 131072 13 8 -0.367908

"C1" "c3" 0 1 131072 14 6 -0.063722

"C2" "c3" 0 1 131072 15 6 0.200863

"O11" "os" 0 1 131072 16 8 -0.513040

"C3" "c3" 0 1 131072 17 6 0.741353

"O12" "oh" 0 1 131072 18 8 -0.852226

"C4" "c3" 0 1 131072 19 6 -0.443178

"C5" "c3" 0 1 131072 20 6 0.265437

"N1" "na" 0 1 131072 21 7 -0.105582

"C6" "c2" 0 1 131072 22 6 0.043665

"N2" "Y1" 0 1 131072 23 7 0.079178

"C7" "c2" 0 1 131072 24 6 -0.534324

"C8" "c2" 0 1 131072 25 6 0.980179

"O13" "o" 0 1 131072 26 8 -0.680644

"N3" "ns" 0 1 131072 27 7 -0.900675

"C9" "c2" 0 1 131072 28 6 1.009076

"N4" "nv" 0 1 131072 29 7 -0.963413

"N5" "n2" 0 1 131072 30 7 -0.812736

"C10" "c2" 0 1 131072 31 6 0.586443

"N6" "Y2" 0 1 131072 32 7 -1.142330

"N7" "Y3" 0 1 131072 33 7 -0.597859

"H1" "h1" 0 1 131072 34 1 0.067284

"H2" "h1" 0 1 131072 35 1 0.067284

"H3" "h1" 0 1 131072 36 1 -0.012167

"H4" "h1" 0 1 131072 37 1 0.098376

"H5" "hc" 0 1 131072 38 1 0.118713

"H6" "hc" 0 1 131072 39 1 0.118713

"H7" "h2" 0 1 131072 40 1 0.076893

"H8" "h5" 0 1 131072 41 1 0.178019

"H9" "hn" 0 1 131072 42 1 0.418070

"H10" "hn" 0 1 131072 43 1 0.292595

"H11" "hn" 0 1 131072 44 1 0.373510

"H12" "hn" 0 1 131072 45 1 0.373510

"H13" "hn" 0 1 131072 46 1 0.382053

"H14" "hn" 0 1 131072 47 1 0.371548

"H15" "ho" 0 1 131072 48 1 0.457877

"Pt1" "M1" 0 1 131072 49 15 0.343746

"H16" "hn" 0 1 131072 50 1 0.371548

"H17" "hn" 0 1 131072 51 1 0.292595

"H18" "hn" 0 1 131072 52 1 0.292595

"Cl1" "Y4" 0 1 131072 53 6 -0.730618

!entry.MOL.unit.atomspertinfo table str pname str ptype int ptypex int pelmnt dbl pchg

"P1" "p5" 0 -1 0.0

"O1" "oh" 0 -1 0.0

"O2" "o" 0 -1 0.0

"O3" "o" 0 -1 0.0

"O4" "os" 0 -1 0.0

"P2" "p5" 0 -1 0.0

"O5" "o" 0 -1 0.0

"O6" "o" 0 -1 0.0

"O7" "os" 0 -1 0.0

"P3" "p5" 0 -1 0.0

"O8" "o" 0 -1 0.0

"O9" "o" 0 -1 0.0

"O10" "os" 0 -1 0.0

"C1" "c3" 0 -1 0.0

"C2" "c3" 0 -1 0.0

"O11" "os" 0 -1 0.0

"C3" "c3" 0 -1 0.0

"O12" "oh" 0 -1 0.0

"C4" "c3" 0 -1 0.0

"C5" "c3" 0 -1 0.0

"N1" "na" 0 -1 0.0

"C6" "cc" 0 -1 0.0

"N2" "Y1" 0 -1 0.0

"C7" "cd" 0 -1 0.0

"C8" "c" 0 -1 0.0

"O13" "o" 0 -1 0.0

"N3" "ns" 0 -1 0.0

"C9" "cd" 0 -1 0.0

"N4" "nv" 0 -1 0.0

"N5" "nc" 0 -1 0.0

"C10" "cc" 0 -1 0.0

"N6" "Y2" 0 -1 0.0

"N7" "Y3" 0 -1 0.0

"H1" "h1" 0 -1 0.0

"H2" "h1" 0 -1 0.0

"H3" "h1" 0 -1 0.0

"H4" "h1" 0 -1 0.0

"H5" "hc" 0 -1 0.0

"H6" "hc" 0 -1 0.0

"H7" "h2" 0 -1 0.0

"H8" "h5" 0 -1 0.0

"H9" "hn" 0 -1 0.0

"H10" "hn" 0 -1 0.0

"H11" "hn" 0 -1 0.0

"H12" "hn" 0 -1 0.0

"H13" "hn" 0 -1 0.0

"H14" "hn" 0 -1 0.0

"H15" "ho" 0 -1 0.0

"Pt1" "M1" 0 -1 0.0

"H16" "hn" 0 -1 0.0

"H17" "hn" 0 -1 0.0

"H18" "hn" 0 -1 0.0

"Cl1" "Y4" 0 -1 0.0

!entry.MOL.unit.boundbox array dbl

-1.000000

0.0

0.0

0.0

0.0

!entry.MOL.unit.childsequence single int

2

!entry.MOL.unit.connect array int

0

0

!entry.MOL.unit.connectivity table int atom1x int atom2x int flags

1 2 1

1 3 1

1 4 1

1 5 1

5 6 1

6 7 1

6 8 1

6 9 1

9 10 1

10 11 1

10 12 1

10 13 1

13 14 1

14 15 1

14 34 1

14 35 1

15 16 1

15 17 1

15 36 1

16 20 1

17 18 1

17 19 1

17 37 1

18 48 1

19 20 1

19 38 1

19 39 1

20 21 1

20 40 1

21 22 1

21 31 1

22 23 2

22 41 1

23 24 1

23 49 1

24 25 1

24 31 2

25 26 2

25 27 1

27 28 1

27 42 1

28 29 1

28 30 2

29 44 1

29 45 1

30 31 1

32 47 1

32 50 1

32 49 1

32 46 1

33 43 1

33 51 1

33 52 1

33 49 1

49 53 1

!entry.MOL.unit.hierarchy table str abovetype int abovex str belowtype int belowx

"U" 0 "R" 1

"R" 1 "A" 1

"R" 1 "A" 2

"R" 1 "A" 3

"R" 1 "A" 4

"R" 1 "A" 5

"R" 1 "A" 6

"R" 1 "A" 7

"R" 1 "A" 8

"R" 1 "A" 9

"R" 1 "A" 10

"R" 1 "A" 11

"R" 1 "A" 12

"R" 1 "A" 13

"R" 1 "A" 14

"R" 1 "A" 15

"R" 1 "A" 16

"R" 1 "A" 17

"R" 1 "A" 18

"R" 1 "A" 19

"R" 1 "A" 20

"R" 1 "A" 21

"R" 1 "A" 22

"R" 1 "A" 23

"R" 1 "A" 24

"R" 1 "A" 25

"R" 1 "A" 26

"R" 1 "A" 27

"R" 1 "A" 28

"R" 1 "A" 29

"R" 1 "A" 30

"R" 1 "A" 31

"R" 1 "A" 32

"R" 1 "A" 33

"R" 1 "A" 34

"R" 1 "A" 35

"R" 1 "A" 36

"R" 1 "A" 37

"R" 1 "A" 38

"R" 1 "A" 39

"R" 1 "A" 40

"R" 1 "A" 41

"R" 1 "A" 42

"R" 1 "A" 43

"R" 1 "A" 44

"R" 1 "A" 45

"R" 1 "A" 46

"R" 1 "A" 47

"R" 1 "A" 48

"R" 1 "A" 49

"R" 1 "A" 50

"R" 1 "A" 51

"R" 1 "A" 52

"R" 1 "A" 53

!entry.MOL.unit.name single str

"MOL"

!entry.MOL.unit.positions table dbl x dbl y dbl z

22.341000 5.247000 4.971000

23.055000 6.361000 5.995000

20.962000 4.891000 5.451000

23.372000 4.180000 4.703000

22.110000 6.242000 3.628000

22.966000 7.350000 2.775000

24.314000 6.805000 2.342000

22.045000 7.973000 1.774000

23.228000 8.438000 4.066000

24.349000 9.623000 4.301000

23.789000 10.483000 5.421000

24.879000 10.221000 3.035000

25.592000 8.739000 5.040000

26.717000 8.324000 4.276000

27.189000 6.925000 4.656000

27.671000 6.856000 6.033000

26.126000 5.824000 4.504000

26.177000 5.239000 3.232000

26.455000 4.847000 5.641000

27.158000 5.721000 6.687000

26.285000 6.202000 7.801000

25.552000 7.349000 7.748000

25.039000 7.669000 8.929000

25.425000 6.666000 9.796000

25.152000 6.438000 11.186000

24.509000 7.058000 12.020000

25.807000 5.220000 11.605000

26.563000 4.392000 10.821000

27.122000 3.270000 11.463000

26.801000 4.606000 9.561000

26.220000 5.750000 9.090000

22.536000 8.954000 7.964000

23.652000 11.417000 8.078000

27.538000 9.044000 4.422000

26.466000 8.300000 3.210000

28.038000 6.673000 4.000000

25.140000 6.247000 4.695000

27.167000 4.096000 5.274000

25.548000 4.349000 5.988000

27.968000 5.193000 7.205000

25.458000 7.935000 6.839000

25.713000 5.039000 12.595000

23.761000 11.244000 7.051000

27.537000 2.681000 10.748000

26.425000 2.740000 11.978000

22.948000 7.252000 5.610000

22.682000 9.201000 6.977000

25.399000 5.661000 2.770000

24.243000 9.590000 8.857000

22.517000 7.933000 7.951000

24.249000 12.173000 8.403000

22.674000 11.602000 8.284000

26.163000 10.770000 10.069000

!entry.MOL.unit.residueconnect table int c1x int c2x int c3x int c4x int c5x int c6x

0 0 0 0 0 0

!entry.MOL.unit.residues table str name int seq int childseq int startatomx str restype int imagingx

"MOL" 1 54 1 "?" 0

!entry.MOL.unit.residuesPdbSequenceNumber array int

0

!entry.MOL.unit.solventcap array dbl

-1.000000

0.0

0.0

0.0

0.0

!entry.MOL.unit.velocities table dbl x dbl y dbl z

0.0 0.0 0.0

0.0 0.0 0.0

0.0 0.0 0.0

0.0 0.0 0.0

0.0 0.0 0.0

0.0 0.0 0.0

0.0 0.0 0.0

0.0 0.0 0.0

0.0 0.0 0.0

0.0 0.0 0.0

0.0 0.0 0.0

0.0 0.0 0.0

0.0 0.0 0.0

0.0 0.0 0.0

0.0 0.0 0.0

0.0 0.0 0.0

0.0 0.0 0.0

0.0 0.0 0.0

0.0 0.0 0.0

0.0 0.0 0.0

0.0 0.0 0.0

0.0 0.0 0.0

0.0 0.0 0.0

0.0 0.0 0.0

0.0 0.0 0.0

0.0 0.0 0.0

0.0 0.0 0.0

0.0 0.0 0.0

0.0 0.0 0.0

0.0 0.0 0.0

0.0 0.0 0.0

0.0 0.0 0.0

0.0 0.0 0.0

0.0 0.0 0.0

0.0 0.0 0.0

0.0 0.0 0.0

0.0 0.0 0.0

0.0 0.0 0.0

0.0 0.0 0.0

0.0 0.0 0.0

0.0 0.0 0.0

0.0 0.0 0.0

0.0 0.0 0.0

0.0 0.0 0.0

0.0 0.0 0.0

0.0 0.0 0.0

0.0 0.0 0.0

0.0 0.0 0.0

0.0 0.0 0.0

0.0 0.0 0.0

0.0 0.0 0.0

0.0 0.0 0.0

0.0 0.0 0.0

File frcmod:

remark gY4es here

MASS

p5 30.970 1.538

oh 16.000 0.465

os 16.000 0.465

c3 12.010 0.878

na 14.010 0.530

c2 12.010 0.360

n2 14.010 0.530

ns 14.010 0.530

nv 14.010 0.530

n9 14.010 0.530

h1 1.008 0.135

hc 1.008 0.135

h2 1.008 0.135

h5 1.008 0.135

hn 1.008 0.161

ho 1.008 0.135

M1 195.08 Pt iY4n

Y1 14.010 0.530

Y2 14.010 0.530

Y3 14.010 0.530

Y4 35.45 1.910

BOND

p5-Y4 529.55 1.487

p5-oh 346.03 1.615

p5-os 346.25 1.615

oh-ho 563.51 0.973

os-c3 284.76 1.432

c3-c3 232.52 1.538

c3-h1 375.92 1.097

c3-oh 293.40 1.423

c3-hc 375.92 1.097

c3-na 262.85 1.463

c3-h2 377.33 1.096

na-c2 327.66 1.401

c2-n2 518.67 1.282

c2-h5 386.13 1.091

c2-c2 481.83 1.334

c2-Y4 635.23 1.225

c2-ns 330.19 1.399 same as c2-n

ns-hn 527.31 1.013 same as hn-n

c2-nv 345.39 1.387 same as c2-nh

nv-hn 529.46 1.012 same as hn-nh

n9-hn 511.28 1.019 same as hn-n3

n2-hn 501.09 1.023

Y4 -ho 540.28 0.981

Y1-M1 80.3 2.1471 Created by SeminariY4 method using MCPB.py

Y2-M1 80.3 2.1471 Created by SeminariY4 method using MCPB.py

Y3-M1 80.0 2.1471 Created by SeminariY4 method using MCPB.py

Y4-M1 212.0 2.10 Created by SeminariY4 method using MCPB.py

Y2-hn 511.28 1.019 same as hn-n3

Y3-hn 501.09 1.023

Y3-ho 501.09 1.023

Y4-ho 540.28 0.981

c2-Y1 518.67 1.282

oh-M1 81.6 2.6178

ANGLE

p5-oh-ho 58.997 110.080

p5-oh-M1 58.997 110.080

p5-Y4 -M1 58.997 110.080

p5-os-p5 106.787 126.250

Y4 -p5-oh 81.901 115.210

Y4 -p5-o 85.510 115.800

Y4 -p5-os 81.819 115.460

oh-p5-os 83.902 101.940

os-p5-os 83.949 101.840

p5-os-c3 83.251 119.540

os-c3-c3 85.306 107.970

os-c3-h1 62.377 109.780

c3-c3-c3 64.888 111.510

c3-c3-h1 46.868 109.560

c3-os-c3 66.293 112.480

c3-c3-oh 84.642 110.190

os-c3-na 109.296 109.030

o -c2-ns 109.296 109.030

os-c3-h2 62.442 109.580

c3-oh-ho 49.027 107.260

c3-c3-hc 46.816 109.800

oh-c3-h1 62.540 110.260

c3-c3-na 82.668 112.880

c3-c3-h2 46.730 110.220

c3-na-c2 65.723 117.200

na-c3-h2 61.963 107.310

na-c2-n2 110.312 123.620

na-c2-h5 59.208 126.390

na-c2-c2 87.232 121.940

c2-na-c2 69.273 110.370

c2-n2-c2 73.202 118.180

n2-c2-h5 64.543 121.700

n2-c2-c2 89.778 126.010

c2-c2-Y4 89.793 130.890

c2-c2-ns 86.693 123.670 same as c2-c2-n

c2-c2-c2 71.539 121.810

c2-ns-c2 67.455 116.750 same as c2-n -c2

c2-ns-hn 47.988 117.900 same as c2-n -hn

Y4 -c2-ns 75.107 117.460 Calculated with empirical approach

ns-c2-nv 113.384 109.350 same as n -c2-nh

ns-c2-n2 110.772 122.820 same as n -c2-n2

c2-nv-hn 48.954 115.090 same as c2-nh-hn

nv-c2-n2 110.708 124.270 same as n2-c2-nh

h1-c3-h1 38.802 108.460

hc-c3-hc 38.960 107.580

hn-nv-hn 39.519 115.120 same as hn-nh-hn

hn-n9-hn 40.828 106.400 same as hn-n3-hn

hn-n2-hn 38.294 120.000

M1-Y2-hn 67.55 104.19 Created by SeminariY4 method using MCPB.py

M1-Y3-hn 67.39 104.19 Created by SeminariY4 method using MCPB.py

M1-Y3-ho 67.39 104.19

M1-Y4-ho 50.01 91.15 Created by Seminario method using MCPB.py

Y1-M1-Y2 182.00 90.94 Created by SeminariY4 method using MCPB.py

Y1-M1-Y3 182.48 180.46 Created by SeminariY4 method using MCPB.py

Y1-M1-Y4 182.50 89.61 Created by SeminariY4 method using MCPB.py

Y2-M1-Y3 182.00 87.62 Created by SeminariY4 method using MCPB.py

Y2-M1-Y4 182.43 183.80 Created by SeminariY4 method using MCPB.py

Y3-M1-Y4 182.71 96.43 Created by SeminariY4 method using MCPB.py

oh-M1-Y2 180.43 83.80 Created by Seminario method using MCPB.py

oh-M1-Y3 182.43 83.80 Created by Seminario method using MCPB.py

oh-M1-Y1 182.43 93.80 Created by Seminario method using MCPB.py

oh-M1-Y4 182.71 86.43 Created by Seminario method using MCPB.py

ho -oh-M1 72.71 86.43 Created by Seminario method using MCPB.py

Y4 -M1-oh 72.71 76.43 Created by Seminario method using MCPB.py

c2-Y1-M1 11.11 120.68 Created by SeminariY4 method using MCPB.py

Y1-c2-c2 89.778 126.010

c2-Y1-c2 73.202 118.180

h5-c2-Y1 52.26 121.70 SOURCE4_SOURCE5 71 2.1538

hn-Y2-hn 40.828 106.400 same as hn-n3-hn

hn-Y3-hn 38.294 120.000

ho-Y3-hn 38.294 120.000

na-c2-Y1 110.312 123.620

ho-Y4-ho 38.294 120.000

DIHE

p5-os-p5-o 1 0.800 0.000 2.000

p5-os-p5-os 1 0.800 0.000 2.000

Y4 -p5-oh-ho 1 0.367 0.000 3.000

oh-p5-os-p5 1 0.800 0.000 2.000

os-p5-oh-ho	1	0.533	0.000	3.000
os-p5-os-c3	1	0.000	0.000	-3.000
os-p5-os-c3	1	2.610	0.000	2.000
p5-os-c3-c3	1	0.383	0.000	-3.000
p5-os-c3-c3	1	3.950	180.000	1.000
p5-os-c3-h1	1	0.217	0.000	3.000
oh-p5-os-c3	1	0.250	0.000	-3.000
oh-p5-os-c3	1	1.200	0.000	2.000
Y4 -p5-os-c3	1	0.800	0.000	-2.000
Y4 -p5-os-c3	1	0.550	0.000	3.000
os-c3-c3-os	1	0.000	0.000	-3.000
os-c3-c3-os	1	0.000	180.000	-2.000
os-c3-c3-os	1	0.170	180.000	1.000
os-c3-c3-c3	1	0.156	0.000	3.000
os-c3-c3-h1	1	0.000	0.000	-3.000
os-c3-c3-h1	1	0.250	0.000	1.000
c3-c3-os-c3	1	0.240	0.000	-3.000
c3-c3-os-c3	1	0.160	0.000	2.000
c3-c3-c3-oh	1	0.210	0.000	3.000
c3-c3-c3-c3	1	0.130	0.000	-3.000
c3-c3-c3-c3	1	0.290	180.000	-2.000
c3-c3-c3-c3	1	0.110	0.000	1.000
c3-c3-c3-h1	1	0.156	0.000	3.000
c3-os-c3-na	1	0.383	0.000	-3.000
c3-os-c3-na	1	0.650	0.000	2.000
c3-os-c3-h2	1	0.383	0.000	3.000
c3-c3-oh-ho	1	0.000	0.000	3.000
c3-c3-c3-hc	1	0.080	0.000	3.000
os-c3-c3-oh	1	1.010	0.000	-3.000
os-c3-c3-oh	1	0.000	0.000	-2.000
os-c3-c3-oh	1	0.020	180.000	1.000
os-c3-c3-hc	1	0.000	0.000	-3.000
os-c3-c3-hc	1	0.250	0.000	1.000
os-c3-na-c2	1	0.000	0.000	-2.000
os-c3-na-c2	1	2.500	0.000	1.000
c3-c3-c3-na	1	0.156	0.000	3.000
c3-c3-c3-h2	1	0.156	0.000	3.000
oh-c3-c3-h1	1	0.000	0.000	-3.000
oh-c3-c3-h1	1	0.250	0.000	1.000
oh-c3-c3-hc	1	0.180	0.000	-3.000
oh-c3-c3-h	1	0.510	0.000	1.000
c3-c3-na-c2	1	0.000	0.000	2.000
c3-os-c3-h1	1	0.337	0.000	3.000
c3-na-c2-n2	1	0.625	180.000	2.000
c3-na-c2-h5	1	0.625	180.000	2.000
c3-na-c2-c2	1	0.625	180.000	2.000
na-c3-c3-hc	1	0.156	0.000	3.000
na-c2-n2-c2	1	4.150	180.000	2.000
na-c2-c2-n2	1	6.650	180.000	2.000
na-c2-c2-c2	1	6.650	180.000	2.000
c2-na-c3-h2	1	0.000	0.000	2.000
c2-na-c2-c2	1	0.625	180.000	2.000
c2-na-c2-n2	1	0.625	180.000	2.000
c2-n2-c2-c2	1	4.150	180.000	2.000
n2-c2-c2-Y4	1	6.650	180.000	2.000
n2-c2-c2-ns	1	6.650	180.000	2.000

n2-c2-c2-n2 1 6.650 180.000 2.000

c2-n2-c2-h5 1 4.150 180.000 2.000

c2-c2-ns-c2 1 0.650 180.000 2.000 same as X -c2-n -X

c2-c2-ns-hn 1 0.650 180.000 2.000 same as X -c2-n -X

c2-c2-c2-n2 1 6.650 180.000 2.000

c2-ns-c2-nv 1 0.650 180.000 2.000 same as X -c2-n -X

c2-ns-c2-n2 1 0.650 180.000 2.000 same as X -c2-n -X

Y4 -c2-c2-c2 1 6.650 180.000 2.000

Y4 -c2-ns-c2 1 0.650 180.000 2.000 same as X -c2-n -X

Y4 -c2-ns-hn 1 0.650 180.000 2.000 same as X -c2-n -X

ns-c2-c2-c2 1 6.650 180.000 2.000

ns-c2-nv-hn 1 0.675 180.000 2.000 same as X -c2-nh-X

ns-c2-n2-c2 1 4.150 180.000 2.000

nv-c2-ns-hn 1 0.650 180.000 2.000 same as X -c2-n -X

nv-c2-n2-c2 1 4.150 180.000 2.000

n2-c2-ns-hn 1 0.650 180.000 2.000 same as X -c2-n -X

n2-c2-nv-hn 1 0.675 180.000 2.000 same as X -c2-nh-X

c2-na-c2-h5 1 0.625 180.000 2.000

h1-c3-c3-h1 1 0.156 0.000 3.000

h1-c3-oh-ho 1 0.113 0.000 3.000

h1-c3-c3-hc 1 0.156 0.000 3.000

hc-c3-c3-h2 1 0.156 0.000 3.000

M1-Y1-c2-c2 3 0.00 0.00 3.0 Treat as zerY4 by MCPB.py

Y1-M1-Y2-hn 3 0.00 0.00 3.0 Treat as zerY4 by MCPB.py

oh-M1-Y2-hn 3 0.00 0.00 3.0 Treat as zero by MCPB.py

oh-M1-Y3-hn 3 0.00 0.00 3.0 Treat as zero by MCPB.py

oh-M1-Y1-c2 3 0.00 0.00 3.0 Treat as zero by MCPB.py

oh-M1-Y4-ho 3 0.00 0.00 3.0 Treat as zero by MCPB.py

Y1-M1-Y3-hn 3 0.00 0.00 3.0 Treat as zerY4 by MCPB.py

Y1-M1-Y4-ho 3 0.00 0.00 3.0 Treat as zero by MCPB.py

Y1-c2-c2-n2 1 6.65 180.0 2.0

Y1-c2-c2-ns 1 6.65 180.0 2.0

Y1-c2-c2-Y4 1 6.65 180.0 2.0

Y2-M1-Y1-c2 3 0.00 0.00 3.0 Treat as zerY4 by MCPB.py

Y2-M1-Y3-hn 3 0.00 0.00 3.0 Treat as zerY4 by MCPB.py

Y2-M1-Y4-ho 3 0.00 0.00 3.0 Treat as zero by MCPB.py

Y3-M1-Y1-c2 3 0.00 0.00 3.0 Treat as zerY4 by MCPB.py

Y3-M1-Y2-hn 3 0.00 0.00 3.0 Treat as zerY4 by MCPB.py

Y3-M1-Y4-ho 3 0.00 0.00 3.0 Treat as zero by MCPB.py

Y4-M1-Y1-c2 3 0.00 0.00 3.0 Treat as zerY4 by MCPB.py

Y4-M1-Y2-hn 3 0.00 0.00 3.0 Treat as zerY4 by MCPB.py

Y4-M1-Y3-hn 3 0.00 0.00 3.0 Treat as zerY4 by MCPB.py

c2-Y1-M1-Y2 3 0.00 0.00 3.0 Treat as zerY4 by MCPB.py

c2-Y1-M1-Y3 3 0.00 0.00 3.0 Treat as zerY4 by MCPB.py

c2-Y1-M1-Y4 3 0.00 0.00 3.0 Treat as zerY4 by MCPB.py

c2-Y1-c2-c2 1 4.15 180.0 2.0

c2-na-c2-Y1 1 0.625 180.0 2.0

c3-na-c2-Y1 1 0.625 180.0 2.0

h5-c2-Y1-M1 3 0.00 0.00 3.0 Treat as zerY4 by MCPB.py

h5-c2-Y1-c2 1 4.15 180.0 2.0

na-c2-Y1-M1 3 0.00 0.00 3.0 Treat as zerY4 by MCPB.py

na-c2-Y1-c2 1 4.15 180.0 2.0

na-c2-c2-Y1 1 6.65 180.0 2.0

ho-Y3-M1-Y4 3 0.00 0.00 3.0

Y2-M1-Y3-ho 3 0.00 0.00 3.0

Y1-M1-Y3-ho 3 0.00 0.00 3.0

o -c2-ns-c2 3 0.00 0.00 3.0

o -c2-ns-hn 3 0.00 0.00 3.0

IMPROPER

c2-c2-na-c3 1.1 180.0 2.0

h5-n2-c2-na 1.1 180.0 2.0 Using default value

c2-c2-c2-n2 1.1 180.0 2.0 Using default value

c2-ns-c2-Y4 1.1 180.0 2.0 Using default value

c2-c2-ns-hn 1.1 180.0 2.0 Using default value

n2-ns-c2-nv 1.1 180.0 2.0 Using default value

c2-hn-nv-hn 1.1 180.0 2.0 Using default value

c2-n2-c2-na 1.1 180.0 2.0 Using default value

Y1-c2-c2-c2 1.1 180.0 2.0 Using default value

Y1-h5-c2-na 1.1 180.0 2.0 Using default value

NONBON

p5 2.0732 0.2295

Y4 1.9452 0.2638

oh 1.8200 0.0930

os 1.7713 0.0726

c3 1.9069 0.1078

na 1.7992 0.2042

c2 1.8606 0.0988

n2 1.8993 0.0941

ns 1.8352 0.1174

nv 1.8903 0.1120

n9 2.2700 0.0095

h1 1.3593 0.0208

hc 1.4593 0.0208

h2 1.2593 0.0208

h5 1.3735 0.0161

hn 0.6210 0.0100

ho 0.3019 0.0047

M1 1.2660 0.0030764200 CM set fY4r Pt2+ ion in TIP3P water from Li et al. JCTC, 2013, 9, 2733

Y1 1.8993 0.0941

Y2 2.2700 0.0095

Y3 1.8993 0.0941
